# Robust control chart for nonlinear conditionally heteroscedastic time series based on Huber support vector regression

**DOI:** 10.1371/journal.pone.0299120

**Published:** 2024-02-23

**Authors:** Chang Kyeom Kim, Min Hyeok Yoon, Sangyeol Lee

**Affiliations:** Department of Statistics, Seoul National University, Seoul, South Korea; Institute for Economic Forecasting, Romanian Academy, ROMANIA

## Abstract

This study proposes a control chart that monitors conditionally heteroscedastic time series by integrating the Huber support vector regression (HSVR) and the one-class classification (OCC) method. For this task, we consider the model that incorporates nonlinearity to the generalized autoregressive conditionally heteroscedastic (GARCH) time series, named HSVR-GARCH, to robustly estimate the conditional volatility when the structure of time series is not specified with parameters. Using the squared residuals, we construct the OCC-based control chart that does not require any posterior modifications of residuals unlike previous studies. Monte Carlo simulations reveal that deploying squared residuals from the HSVR-GARCH model to control charts can be immensely beneficial when the underlying model becomes more complicated and contaminated with noises. Moreover, a real data analysis with the Nasdaq composite index and Korea Composite Stock Price Index (KOSPI) datasets further disclose the validity of using the bootstrap method in constructing control charts.

## 1 Introduction

In this study, we develop a statistical process control (SPC) method for a nonlinear conditionally autoregressive heteroscedastic (GARCH) time series whose underlying structure is unspecified, possibly possessing noises. SPC has been developed during the past decades to improve the quality of products through the monitor and control of manufacturing processes [1]. Generally, control charts are appreciated as predominant devices that utilize graphical descriptions of the characteristics of a process, which is designed to promptly signal the out-of-control state of processes. The conventional charts are originally built for independent or uncorrelated observations, but these do not work adequately when they are correlated over time as the correlations among observations cause a high rate of false alarms, see [2–7]. As a remedy, control charts that monitor dependent observations have been proposed in the literature, see [8–11], and further refer to [12] for more recent development.

In practice, however, constructing control charts to monitor GARCH-type time series can be quite challenging, especially when financial time series is targeted. This is so because in financial time series, the task of finding an elongated period of in-control state observations is not feasible, as the time series occasionally suffers from structural instabilities attributed to external socioeconomic factors. This implies that financial time series can become nonstationary if observed for an extended amount of time, and it is difficult to single out a specific model due to the unpredictability of such economic affairs. Although variants of parametric GARCH models were coined to estimate time-varying volatilities, they are commonly apt to violate the stationarity assumptions, needed for precisely estimating model parameters [13]. In practice, accurately calculating residuals based on the presumed models is critical as they are directly harnessed in the construction of the control charts, and therefore, the misspecification of underlying models can be a serious issue in designing control charts. To alleviate this problem, practitioners have developed nonparametric machine learning-based control charts. For an overview, we refer to [14, 15]. Among those, we pay special attention to one-class classification (OCC)-based control charts, which are designed to identify the boundary of a specific class among all data points via learning from a training dataset. For relevant works, we refer to [16–18].

To design a flexible model that meticulously captures the structure of the conditional volatility and avoid the model assumptions, we root our method to the support vector regression (SVR), a variant of the support vector machine (SVM), revised for regression. As SVR can afford to approximate the nonlinearity between variables without knowing their underlying dynamic structure a priori, while concurrently implementing the structural risk minimization principle [19] that balances between the model complexity and the empirical risk [20], SVR has been accepted as a promising tool to estimate the volatility of time series. For a general overview of SVR, we refer to [21–23], and further refer to [24–26] for incorporating SVR to estimate time series models. Specifically, [27, 28] used the extended GARCH model, named SVR-GARCH, to obtain residuals and construct both retrospective change point tests and monitoring methods.

However, as GARCH-type time series inherently suffers from instability as observations can become wildly explosive, monitoring methods that accommodate variants of parametric- or SVR-GARCH models are often inefficient for constructing a reliable test. For instance, [29] fitted an AR(*p*) model to the obtained residuals and truncated excessively large residuals to prevent them from undermining the Type I error rate of the test. This phenomenon arises primarily because standard SVRs still can be susceptible to the outliers lying in the training dataset [30], which generally makes the residuals behave in a more correlated manner, while also rendering some of them to become excessively larger than the others. This deficiency can be severely detrimental against the accuracy of monitoring as it requires “well-behaved” in-control observations or residuals as a primary ingredient of time series monitoring.

To circumvent the problem, we integrate the robust variant of SVR to estimate nonlinear GARCH-type time series models. Among the variants of robust SVR in the literature, such as [31, 32], we specifically employ the Huber SVR (HSVR) by [33] when estimating nonlinear GARCH models. HSVR not only allows to design more flexible models by providing methods to incorporate asymmetry, but also it is suitable for efficiently fitting GARCH models as it suppresses the effects of the explosive observations. Therefore, we also adopt the nonlinear GARCH model using HSVR, called the HSVR-GARCH model, to accommodate a broad class of nonlinear GARCH models.

Moreover, to facilitate the control chart to effectively reflect the sophisticated structure of time series, we propose a control chart that harnesses a nonparametric data description method. In particular, we utilize the support vector data description (SVDD) method, a version of SVM reconstructed to solve the OCC problems [34, 35]. SVDD provides a hypersphere boundary on the data points, aimed to contain the desired proportion of observations within that hypersphere of a minimum radius, which can detect the data point out of the hypersphere boundary as an anomaly. This approach has been taken by several authors and proved to be useful in practice [16, 36–39]. More recently, [40] considered a control chart based on the variant of one-class SVM that well detects mean shifts of a given process. Herein, we utilize squared residuals to train SVDD and determine the decision boundary of in-control time series as a control limit.

The rest of this paper is organized as follows. Section 2 summarizes two variants of SVR, namely, HSVR and SVDD. Section 3.1 elaborates the specification and the process of fitting HSVR-GARCH model alongside with its advantages, while Section 3.2 enumerates the procedure to train the OCC-based control chart using SVDD. Section 4 conducts Monte Carlo simulations to evaluate the performance of control charts that utilize HSVR-GARCH residuals in various circumstances, including the case that observations contain innovational or additive outliers. Our simulation experiments also evaluate the performance of control charts with HSVR-GARCH residuals when they are trained with the samples generated through a bootstrap method. Section 5 performs a real data analysis using the Nasdaq composite index and the Korean stock price index (KOSPI) to further solidify the validity of using bootstrap samples obtained from HSVR-GARCH models. Finally, Section 6 provides the concluding remarks.

## 2 Machine learning methods

This section introduces two machine learning methods, namely, the HSVR-GARCH model and the support vector data description (SVDD) method that comprise the control chart for monitoring nonlinear GARCH-type time series. We first provide a concise overview of these learning methods and then elaborate the procedure to apply those in constructing our monitoring schemes in the subsequent sections.

### 2.1 Huber SVR

Support vector regression (SVR) is a statistical learning method that originated from [41], which aims to precisely estimate the structure of a given dataset nonparametrically. Unlike many traditional parametric time series models, the strength of estimating time series model using SVR arises from its innate versatility to model dataset whose underlying structure is unknown or contains a severe degree of nonlinearity. This strength is particularly beneficial especially when estimating the structure of time series with heteroscedastic volatility, such as financial indices, as conditional volatility is unobservable in general and can be complex due to the interdependent nature of the current global economy. Moreover, utilizing SVR to estimate the structure of the time series can help to simultaneously avoid deploying a sophisticated parametric model, such as the family of exponential GARCH (EGARCH) models [42].

In this study, we utilize the Huber SVR (HSVR) when modeling nonlinear GARCH models to escalate the robustness. HSVR is advantageous over the standard SVR as it can effectively offset the effects of sporadic outliers, frequently observed in time series. Although many versions regarding HSVR exist in the literature, we utilize the *ϵ*-insensitive HSVR proposed by [33, 43].

Given a set of training observations {(**x**_*i*_, *y*_*i*_)} (*i* = 1, …, *n*, ${\text{x}}_{i}\in R^{m},y_{i}\in R$), HSVR seeks to find a function of the form:

$$\begin{array}{c} f\left(\text{x}\right)=K_{1}\left(\text{x},{\text{A}}^{T}\right)\text{w}+b, \end{array}$$ (1)

where $\text{x}\in R^{m}$, $\text{A}\in R^{n\times m}$ is a matrix whose *j*-th row consists of ${\text{x}}_{j}^{T}$ (*j* = 1, …, *n*), $\text{w}\in R^{n}$ and b∈R are parameters to be estimated, and $K_{1}\left(\text{x},{\text{A}}^{T}\right)=\left(K_{1}\left(\text{x},{\text{x}}_{1}\right),\cdots ,K_{1}\left(\text{x},{\text{x}}_{n}\right)\right)\in R^{n}$ with *K*_1_(**x**_1_, **x**_2_) = 〈*ϕ*_1_(**x**_1_), *ϕ*_1_(**x**_2_)〉 for some predefined kernel *K*. Here, 〈⋅, ⋅〉 and *ϕ*_1_(**x**) respectively denote the inner product of the feature space and the implicit kernel operator corresponding to the kernel *K*_1_.

At first glance, (1) seems to deviate from the function form of the standard SVR, which is given as,

$$\begin{array}{c} g\left(\text{x}\right)≔\left\langle \text{w},\phi \left(\text{x}\right)\right\rangle +b=\sum_{j=1}^{n}w_{j}K\left(\text{x},{\text{x}}_{j}\right)+b, \end{array}$$ (2)

where *K* and *ϕ* is defined analogously to *K*_1_ and *ϕ*_1_, respectively. However, we can see that (1) and (2) are actually identical, which implies that HSVR can be viewed as a direct extension of the standard SVR. This is because by using the definition of *K*_1_(**x**, **A**^*T*^), (1) can also be rewritten as

$$\begin{array}{c} f\left(\text{x}\right)=\sum_{j=1}^{n}w_{j}K_{1}\left(\text{x},{\text{x}}_{j}\right)+b, \end{array}$$

where *w*_*j*_ is the *j*-th element of the vector **w**. Therefore, analogous to SVR, estimating (1) using HSVR can be understood as implicitly performing the following two-stage procedure: (i) first map the given observations (**x**_1_, ⋯, **x**_*n*_) onto some feature space using the prescribed mapping function *ϕ*_1_ to produce (**z**_1_, ⋯, **z**_*n*_) ≔ (*ϕ*(**x**_1_), ⋯, *ϕ*(**x**_*n*_)), (ii) then estimate the linear function on that feature space that best describes the mapped observations **z**_*i*_. For more details, we refer to [20, 28] for the standard SVR and [33] for HSVR.

**Fig 1:**
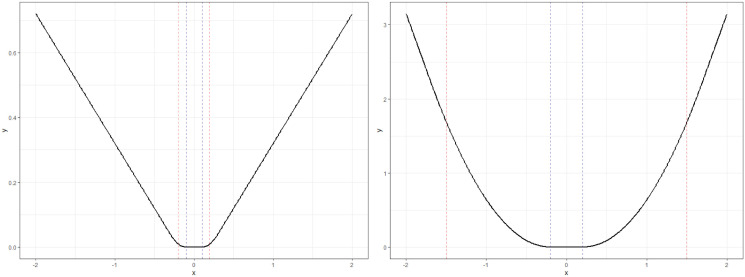
A plot of *ϵ*-insensitive Huber loss function in (4) for (*ϵ*, *γ*) = (0.1, 0.2) (left) and (*ϵ*, *γ*) = (0.5, 1.5) (right).

To estimate the model parameters **w** and *b* in (1), we solve

$$\begin{array}{c} \underset{\left(\text{w},b\right)\in R^{n+1}}{\text{argmin}}\;\frac{1}{2}({\text{w}}^{T}\text{w}+b^{2})+\frac{C_{1}}{2}\sum_{i=1}^{n}L(y_{i}-f({\text{x}}_{i})), \end{array}$$ (3)

where *C*_1_ is a tuning parameter that balances between the model risk and the flatness of the estimated function, and *L* is an *ϵ*-insensitive Huber loss function defined as follows:

$$\begin{array}{c} L\left(x\right)={\left(x-\epsilon \right)}_{+}^{2}+{\left(-x-\epsilon \right)}_{+}^{2}-(x-\epsilon -\gamma {)}_{+}^{2}-(-x-\epsilon -\gamma {)}_{+}^{2}, \end{array}$$ (4)

where *x*_+_ ≔ max(*x*, 0) for x∈R, and *ϵ*, *γ* ≥ 0 are tuning parameters that determine the degree of robustness, see Fig 1 for an illustration.

As depicted in Fig 1, *ϵ*-insensitive Huber loss function returns zero when *x* ∈ [−*ϵ*, *ϵ*], increases quadratically in (−*γ*, −*ϵ*] and (*ϵ*, *γ*], then increases linearly otherwise. Moreover, in a statistical perspective, the optimization problem (3) attains the shrinkage estimator regarding **w** and *b* rather than only **w**, as seen in the traditional SVR models. This alteration is employed to derive a unique solution of **w** and *b* in solving (3) while simultaneously maintaining the strong convexity, refer to [44].

Despite the similarities between HSVR and SVR, the former deviates from the latter in terms of the way of solving the given optimization problem. Indeed, HSVR directly solves the primal problem by taking an iterative approach. To attain the solution, we first explicitly expand (3) by substituting (4), then reparametrize the problem with respect to **z** ≔ (**w**^*T*^, *b*)^*T*^:

$$\begin{array}{c} \begin{array}{cc} \underset{z\in R^{n+1}}{\text{min}}\;L\left(\text{z}\right)= & \frac{1}{2}{\text{z}}^{T}\text{z}+\frac{C_{1}}{2}[\|(\text{y}-\text{Bz}-\epsilon 1_{n}{)}_{+}{\|}^{2}+\|(\text{Bz}-\text{y}-\epsilon 1_{n}{)}_{+}{\|}^{2}\\ & -\|{\left(\text{y}-\text{Bz}-\left(\epsilon +\gamma \right)1_{n}\right)}_{+}{\|}^{2}-\|{\left(\text{Bz}-\text{y}-\left(\epsilon +\gamma \right)1_{n}\right)}_{+}{\|}^{2}], \end{array} \end{array}$$ (5)

where **y** = (*y*_1_, …, *y*_*n*_)^*T*^, **B** = [**K**_1_ **1**_*n*_], **K**_1_ is an *n* × *n* matrix, whose (*i*, *j*)-th element is **K**_1,*ij*_ = *K*_1_(**x**_*i*_, **x**_*j*_), **A** is a matrix of explanatory variables, whose *i*-th row is defined as ${\text{x}}_{i}^{T}$, and $1_{n}\in R^{n}$ is a vector of ones. As the objective function is strongly convex with respect to **z**, we attain the optimality by obtaining the solution of the first-order condition, that is,

$$\frac{\partial L\left(\text{z}\right)}{\partial \text{z}}=0.$$

Therefore, (5) reduces to the problem of the following iterative method that computes the updated solution **z**_*i*+1_ from the previous iteration **z**_*i*_:

$$\begin{array}{c} \begin{array}{cc} {\text{z}}_{i+1}= & {\left(\text{diag}\left(C_{1}^{-1}1_{n}\right)+{\text{B}}^{T}\text{B}\right)}^{-1}{\text{B}}^{T}[\text{y}+1_{n}+\frac{\left|\text{y}-\text{B}{\text{z}}_{i}-\epsilon 1_{n}|-|\text{B}{\text{z}}_{i}-\text{y}-\epsilon 1_{n}\right|}{2}\\ & +{\left(\text{B}{\text{z}}_{i}-\text{y}-\left(\epsilon +\gamma \right)1_{n}\right)}_{+}-{\left(\text{y}-\text{B}{\text{z}}_{i}-\left(\epsilon +\gamma \right)1_{n}\right)}_{+}] \end{array} \end{array}$$

for *i* = 0, 1, …. The general process of using the aforementioned HSVR for GARCH models is presented in Section 3.

### 2.2 Support vector data description algorithm

To formulate the control chart that integrates the one-class classification algorithm, we use the support vector data description (SVDD) model in this study, which is a complex of support vector machine (SVM) and the data description algorithm proposed by [35, 45]. SVDD is a method to find a boundary that encompasses given observations with a single hypersphere in the feature space. Because the proportion of observations inside the hypersphere can be settled in advance, we can readily employ SVDD as an anomaly detection method. For a general overview, we refer to [36].

The fundamental concept of SVDD stems from constructing a hypersphere with a minimum possible volume that includes the desired number of observations within such a boundary. Namely, given the training dataset ${\text{x}}_{i}\in R^{p}$ (*i* = 1, …, *n*), SVDD solves the following optimization problem with respect to the center $\text{a}\in R^{p}$ and the radius *R* ≥ 0 of the hypersphere:

$$\begin{array}{c} \begin{array}{cc} \text{minimize} & R^{2}+C_{2}\sum_{i=1}^{n}\xi_{i}\\ \text{subject}\;\text{to} & \left|\right|\phi_{2}\left({\text{x}}_{i}\right)-\text{a}{\left|\right|}^{2}\leq R^{2}+\xi_{i}, \end{array} \end{array}$$ (6)

where *ξ*_*i*_ ≥ 0 (*i* = 1, ⋯, *n*) denotes a slack variable that penalizes observations lying outside the hypersphere, *C*_2_ ≥ 0 is a tuning parameter which controls the balance between the misclassification errors and the volume of the hypersphere, and *ϕ*_2_(**x**) is an implicit kernel operator that is defined analogously to the kernel function *K*_1_ in Section 2.1, namely,

$$K_{2}\left({\text{x}}_{i},{\text{x}}_{j}\right)=\left\langle \phi_{2}\left({\text{x}}_{i}\right),\phi_{2}\left({\text{x}}_{j}\right)\right\rangle .$$

In practice, the following Gaussian kernel function is widely selected:

$$\begin{array}{c} K_{2}\left({\text{x}}_{i},{\text{x}}_{j}\right)=\text{exp}\left(-\frac{\left|\right|{\text{x}}_{i}-{\text{x}}_{j}{\left|\right|}^{2}}{2\kappa^{2}}\right), \end{array}$$ (7)

where *κ* > 0 is a tuning parameter that determines the complexity of the decision boundary. In practice, the decision boundary becomes more nonlinear and sophisticated when *κ* is small.

The general procedure of solving (6) is similar to that of the classical SVM. To elaborate, we first construct the unconstrained problem as below:

$$\begin{array}{c} L\left(R,a,\alpha_{i},\gamma_{i},\xi_{i}\right)=R^{2}+C_{2}\sum_{i=1}^{n}\xi_{i}-\sum_{i=1}^{n}\alpha_{i}\{R^{2}+\xi_{i}-\left(|\right|\phi_{2}\left({\text{x}}_{i}\right)-{\text{a}||}^{2})\}-\sum_{i=1}^{n}\gamma_{i}\xi_{i}, \end{array}$$ (8)

where *α*_*i*_ ≥ 0 and *γ*_*i*_ ≥ 0 (*i* = 1, …, *n*) are Lagrange multipliers. Then, by using the Karush-Khun-Tucker conditions, we obtain the following equivalent dual problem with respect to *α*_*i*_:

$$\begin{array}{c} \begin{array}{cc} \text{maximize} & \sum_{i=1}^{n}\alpha_{i}K_{2}\left({\text{x}}_{i},{\text{x}}_{i}\right)-\sum_{i,j=1}^{n}\alpha_{i}\alpha_{j}K_{2}\left({\text{x}}_{i},{\text{x}}_{j}\right)\\ \text{subject}\;\text{to} & 0\leq \alpha_{i}\leq C_{2}\;\left(i=1,\ldots ,n\right),\\ & \sum_{i=1}^{n}\alpha_{i}=1. \end{array} \end{array}$$ (9)

Notice that *γ*_*i*_ vanishes due to the relationship *α*_*i*_ = *C*_2_ − *γ*_*i*_, (*i* = 1, …, *n*), which is obtainable using the first-order conditions. Using the optimal *α*_*i*_ of the problem (9), the radius *R*^2^ can be calculated by the formula:

$$\begin{array}{c} R^{2}=K_{2}\left({\text{x}}_{sv},{\text{x}}_{sv}\right)-2\sum_{i=1}^{n}\alpha_{i}K_{2}\left({\text{x}}_{sv},{\text{x}}_{i}\right)+\sum_{i,j=1}^{n}\alpha_{i}\alpha_{j}K_{2}\left({\text{x}}_{i},{\text{x}}_{j}\right), \end{array}$$

where **x**_*sv*_ denotes one of the observations that is precisely located on the boundary of the constructed hypersphere. Similarly, we measure the kernel distance between a new observation $\text{z}\in R^{p}$ and the center **a** by computing

$$\begin{array}{c} D^{2}\left(\text{z}\right)=K_{2}\left(\text{z},\text{z}\right)-2\sum_{i=1}^{n}\alpha_{i}K_{2}\left(\text{z},{\text{x}}_{i}\right)+\sum_{i,j=1}^{n}\alpha_{i}\alpha_{j}K_{2}\left({\text{x}}_{i},{\text{x}}_{j}\right), \end{array}$$ (10)

and **z** is classified as an anomaly when *D*^2^ is larger than *R*^2^.

A primary reason why SVDD can be deployed in constructing a control chart is the usage of kernels, which enables practitioners to design a tailored control chart suitable to their needs. Indeed, the control limit of the chart can be determined by the tuning parameter *C*_2_ in (6), as it can be exploited to precisely specify the rate of the misclassified datasets. To illustrate, by adjusting *C*_2_, we can reset the upper bound *ρ* > 0 of the misclassification error rate through the following relationship:

$$\begin{array}{c} \rho =\frac{1}{nC_{2}}, \end{array}$$ (11)

and it is known that *ρ* matches the misclassification rate when the number of observations are sufficiently large and the distribution of the dataset is continuous [45]. Control charts using SVDD can be remarkably favorable when the underlying structure of the dataset is sophisticated, as SVDD seeks the boundary from a higher-dimensional feature space that can be further adjusted using *κ* in (7). Therefore, tuning *C*_2_ is analogous to adjusting the control limit of control charts, see Sections 3 and 4 for more details.

## 3 OCC-based monitoring scheme

In this section, we summarize the general procedure of constructing the OCC-based control chart. The procedure is divided into two parts: first fitting the HSVR-GARCH model to obtain residuals, then deploying SVDD to determine the control limit.

### 3.1 HSVR-GARCH model

We extend the GARCH model to capture the nonlinear structure of the conditional volatility specified as follows:

$$\begin{array}{c} \left\{ \begin{array}{c} y_{t}=\sigma_{t}\epsilon_{t},\\ \sigma_{t}^{2}=f\left(y_{t-1}^{2},\sigma_{t-1}^{2}\right), \end{array}\right. \end{array}$$ (12)

where *f* is an unknown nonlinear function with the form in (1), which would be obtained by solving (3), $\sigma_{t}^{2}$ is the conditional volatility at time *t* = 1, …, *n*, and *ϵ*_*t*_ are independent and identically distributed (iid) errors with *E*(*ϵ*_1_) = 0 and Var(*ϵ*_1_) = 1. Observe that, as $\sigma_{t}^{2}$ is a (conditional) variance, both $\sigma_{t}^{2}$ and *σ*_*t*_ must be nonnegative by definition.

Model (12) includes a broad class of stationary GARCH time series models. For a class of parametric GARCH models, [46] studied the estimation of their parameters and presented a method of conducting the change point test as an application. Their research later offered a theoretical foundation that based the OCC control charts of [47].

Although it seems plausible to directly deploy HSVR to model obtain the function *f* in (12), it is actually impractical because HSVR does not automatically guarantee the conditional variance $\sigma_{t}^{2}$ to be nonnegative. To rectify the problem, we modify the above model by taking the logarithm to ensure the nonnegativity of $\sigma_{t}^{2}$ as follows:

$$\begin{array}{c} \left\{ \begin{array}{cc} y_{t} & =\sigma_{t}\epsilon_{t},\\ \text{log}\;\sigma_{t}^{2} & =g\left(y_{t-1}^{2},\sigma_{t-1}^{2}\right). \end{array}\right. \end{array}$$ (13)

As $\sigma_{t}^{2}$ is not observable in most practical circumstances when estimating *g* in (13), we substitute $\sigma_{t}^{2}$ in (12) with

$$\begin{array}{c} {\tilde{\sigma }}_{t}^{2}=\frac{1}{s}\sum_{i=0}^{s-1}y_{t-i}^{2} \end{array}$$ (14)

for *t* = 1, …, *n*, and *s* ≥ 1 denotes a degree of smoothness. When *t* < *s*, (14) is defined as $\sigma_{t}^{2}≔\frac{1}{t}\sum_{i=0}^{t-1}y_{t-i}^{2}$. ${\tilde{\sigma }}_{t}^{2}$ is one of the most widely accepted proxy of $\sigma_{t}^{2}$ and can be readily deployed when predicting the conditional volatility [48] and constructing monitoring schemes [28]. In this study, we set *s* = 20, as it renders the ${\hat{\sigma }}_{t}^{2}$ to be stabilized around the level of the unconditional variance, thereby enhancing the quality of residuals utilized in the formulation of control charts.

Another repercussion due to the unknown $\sigma_{t}^{2}$ is that an adequate source, purposed to compare with ${\hat{\sigma }}_{t}^{2}$ for computing the empirical loss, is absent when aiming to determine the optimal set of tuning parameters, as ${\hat{y}}_{t}$ is always estimated to be zero for pure GARCH-type models. This definitely prohibits practitioners from using standard loss functions, such as the mean absolute error (MAE), when performing the tuning parameter optimization. We overcome this problem by introducing a likelihood-based loss *ℓ*(*g*) for a function *g* with a regularization term regarding **w**. Specifically, by temporarily assuming that the error term *ϵ*_*t*_ is normally distributed, we consider the negative log-likelihood of *g* in (13) as follows:

$$\begin{array}{c} \frac{1}{n}\sum_{t=1}^{n}[\frac{y_{t}^{2}}{\sigma_{t}^{2}}+\text{log}\;\sigma_{t}^{2}]=\frac{1}{n}\sum_{t=1}^{n}[\frac{y_{t}^{2}}{e^{\left\{g\left({\text{x}}_{t}\right)\right\}}}+g\left({\text{x}}_{t}\right)] \end{array}$$

with ${\text{x}}_{t}={\left(y_{t-1}^{2},\sigma_{t-1}^{2}\right)}^{T}$. Then, by appending the regularization term regarding **w** to prevent overfitting, we obtain the following loss function [49]:

$$\begin{array}{c} \ell \left(g\right)=\sum_{t=1}^{n}[y_{t}^{2}e^{-g\left({\text{x}}_{t}\right)}+g\left({\text{x}}_{t}\right)]+\frac{\delta }{2}{\left\|\text{w}\right\|}^{2}, \end{array}$$ (15)

where *δ* ≥ 0 is a tuning parameter that balances between the model’s flatness and the goodness of fit. This approach is conceptually similar to that of the quasi maximum-likelihood estimator (QMLE), which is the standard method of estimation in parametric GARCH models [13].

The general procedure to fitting a HSVR-GARCH model is based on the sequential *k*-fold cross-validation method for time series. To elaborate, we follow the steps below:

**Step 1**. Partition the time series (**x**_*t*_, *y*_*t*_) into chunks of *k* ≥ 2, and denote the time series of the *m*-th chunk as $\left({\text{x}}_{t}^{\left(m\right)},y_{t}^{\left(m\right)}\right)$ (*m* = 1, ⋯, *k*). Moreover, let *n*_*m*_ be the number of observations in the chunk *m*.
**Step 2**. Use the first *m* − 1 chunks to obtain the estiamted function $\hat{g}\left(\text{z}\right)≔K_{1}\left(\text{z},{\text{A}}^{T}\right)\hat{\text{w}}+\hat{b}$ and the estimated conditional volatility in (13) as ${\hat{\sigma }}_{t}^{2}=\text{exp}\left(\hat{g}\left(\text{z}\right)\right)$.
**Step 3**. Using the *m*-th chunk, compute the loss in (15) for the *m*-th chunk and denote the result as $\ell_{m}\left(\hat{g}\right)$, namely,
  $$\begin{array}{c} \ell_{m}\left(\hat{g}\right)≔\sum_{t=1}^{n_{m}}[{\left(y_{t}^{\left(m\right)}\right)}^{2}e^{-{\left({\text{x}}_{t}^{\left(m\right)}\right)}^{2}}+\hat{g}\left({\text{x}}_{t}^{\left(m\right)}\right)]+\frac{\delta }{2}{\left\|\hat{\text{w}}\right\|}^{2}. \end{array}$$
**Step 4**. Repeat Steps 2 and 3 for *m* = 2, ⋯, *k*.
**Step 5**. The loss function that determines the optimal tuning parameter is finally defined as
  $$\ell^{*}\left(\hat{g}\right)≔\frac{1}{k-1}\sum_{m=2}^{k}\ell_{m}\left(\hat{g}\right),$$
  and the best set of tuning parameters is then set to those that minimize $\ell^{*}\left(\hat{g}\right)$.

This process of obtaining ${\hat{\sigma }}_{t}^{2}$ via HSVR-GARCH models is encapsulated in Algorithm 1 below.

The set of tuning parameters for HSVR-GARCH model consist of four elements: (i) the regularization parameter *C* in (3), (ii) two parameters that comprise the *ϵ*-insensitive Huber-loss function *ϵ* and *γ*, (iii) the kernel tuning parameter *s*^2^ for the Gaussian kernel in Section 2.1

$$\begin{array}{c} K_{1}\left({\text{x}}_{1},{\text{x}}_{2}\right)=\text{exp}\left(-\frac{\left|\right|{\text{x}}_{1}-{\text{x}}_{2}{\left|\right|}^{2}}{2s^{2}}\right), \end{array}$$ (16)

and (iv) the regularization parameter *δ* for the likelihood-based loss (15). In this study, we initially fix *δ*, then seek for the remaining tuning parameters that minimize $L\left(\hat{g}\right)$ via the particle swarm optimization. The specifications regarding the tuning parameters are presented in Sections 4 and 5 below.

**Fig 2:**
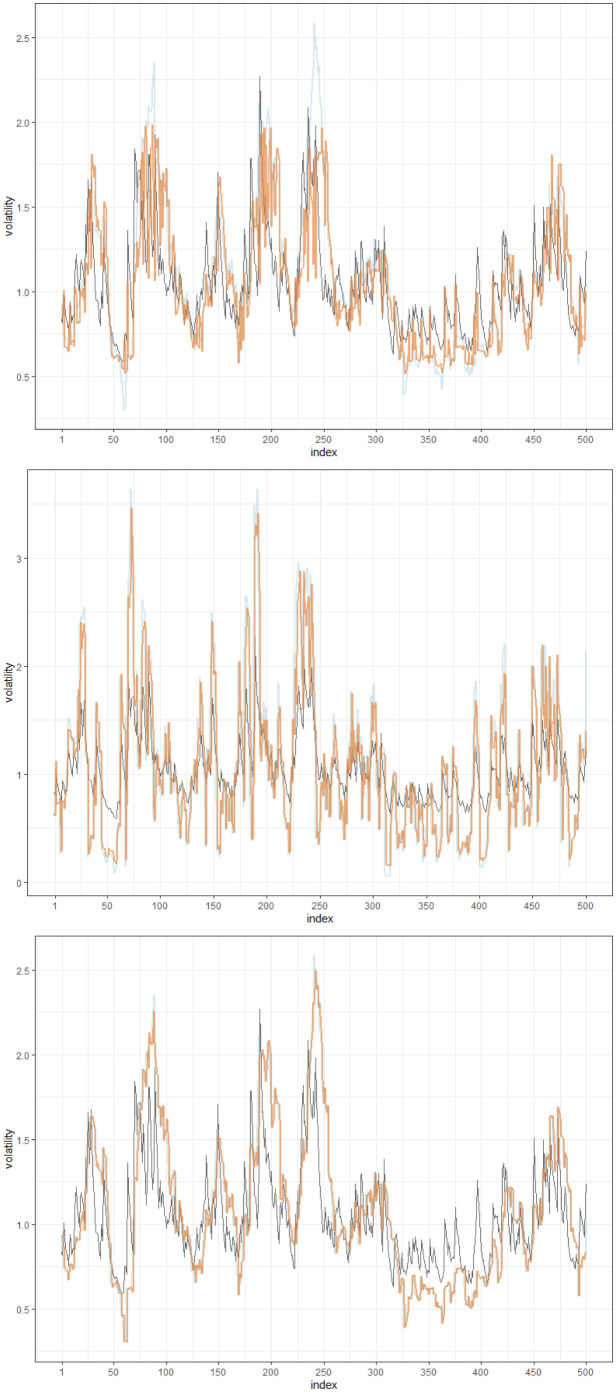
Plot of the estimated conditional volatility obtained from fitting HSVR-GARCH with *s* = 20 (top) and SVR-GARCH models with *s* = 5 (center), 20 (bottom), respectively, when the underlying model is GARCH(1,1) in (17) with (*ω*, *α*, *β*) = (0.1, 0.1, 0.8). Orange and black lines respectively denote the estimated (${\hat{\sigma }}_{t}^{2}$) and true volatility ($\sigma_{t}^{2}$), while the translucent blue line depicts ${\tilde{\sigma }}_{t}^{2}$ with the specified *s*.

One of the prominent advantages of utilizing the HSVR-GARCH model over the SVR-GARCH model of [27] is that it significantly stabilizes the estimated volatility, thus liberates from the necessity of any posterior treatment of residuals when constructing the control chart. Due to the unpredictable nature of GARCH models, conditional volatility is prone to have abrupt anomalies, even when no structural breaks exist. As such, one of the innate drawbacks of the SVR-GARCH model is that it occasionally returns incorrectly estimated conditional variance, as witnessed in Fig 2, which results in the underestimation of $\sigma_{t}^{2}$ in regions where the time series is relatively stable. This may prevent practitioners from directly utilizing the residuals based on the SVR-GARCH model because some residuals are computed to be explosively large. This phenomenon might have occurred as the SVR-GARCH method uses the mean absolute error-type loss as shown below that compares the estimated ${\hat{\sigma }}_{t}^{2}$ with the proxy ${\tilde{\sigma }}_{t}^{2}$ in (14) in optimizing tuning parameters:

$$\begin{array}{c} \ell_{MAE}\left({\hat{\sigma }}_{t}^{2}\right)=\frac{1}{m-k}\sum_{t=k+1}^{m}\left|{\hat{\sigma }}_{t}^{2}-{\tilde{\sigma }}_{t}^{2}\right|, \end{array}$$

where *k* and *m* respectively denote the length of the training and the total length of the time series. In fact, two aforementioned figures concurrently depict that ${\hat{\sigma }}_{t}^{2}$ estimated from the SVR-GARCH model does not heavily depart from ${\tilde{\sigma }}_{t}^{2}$, and fails to resemble the true $\sigma_{t}^{2}$ in some occasions. On the other hand, by employing the likelihood-based loss in (15) with an optimal set of tuning parameters, the HSVR-GARCH method can escape from comparing ${\hat{\sigma }}_{t}^{2}$ against the proxy variables which were already deployed in obtaining $\hat{g}$. This alteration substantially stabilizes the estimation process producing ${\hat{\sigma }}_{t}^{2}$, which more closely resembles the true $\sigma_{t}^{2}$, as reflected in Fig 2, and thereby, results in producing well-behaved residuals.

**Algorithm 1** HSVR-GARCH

 **Input**: A time series ${\left\{y_{t}\right\}}_{t=1}^{n}$, a kernel *K*_1_,

    a collection of tuning parameters A,s≥1,k≥2

 **Output**: A conditional volatility estimator $\hat{g}$

1: Set 1 ≤ *n*_*i*_ ≤ *n* (*i* = 1, …, *k*), *m*_0_ ≡ 0, *n*_*k*_ ≡ *n*, *n*_*i*_ < *n*_*j*_ (*i* < *j*)

2: ${\text{Y}}_{n_{i}}\leftarrow \left\{y_{n_{i-1}+1},\ldots ,y_{n_{i}}\right\}$ (*i* = 1, …, *k*)

3: **for** each pair of z∈A **do**

4:  **for** 2 ≤ *i* ≤ *k* **do**

5:   ${\text{Y}}_{train}\leftarrow \cup_{j=1}^{i-1}{\text{Y}}_{n_{j}}$

6:   With **Y**_*train*_,

7:    ${\tilde{\sigma }}_{t}^{2}\leftarrow \sum_{k=1}^{s}y_{t-k+1}^{2}/\left(s\vee t\right)\;\;\left(1\leq t\leq n_{i-1};\;y_{l}\equiv 0\;\text{for}\;l\leq 0\right)$

8:    ${\text{x}}_{i}\leftarrow {\left(y_{t-1}^{2},{\tilde{\sigma }}_{t-1}^{2}\right)}^{T}$; $\text{A}\leftarrow \left({\text{x}}_{1},{\text{x}}_{2},\ldots ,{\text{x}}_{n_{i}}\right)$

9:    *g*_**z**_ ← *K*_1_(**x**_*i*_, **A**^*T*^)**w**_**z**_ + *b*_**z**_

10:    ${\hat{g}}_{\text{z},i}\leftarrow {\text{argmin}}_{\left({\text{w}}_{\text{z}},b_{\text{z}}\right)}\left({\text{w}}_{\text{z}}^{T}{\text{w}}_{\text{z}}+b_{\text{z}}^{2}\right)/2+C\sum_{i=1}^{n}L\left(y_{i}-g_{\text{z}}\left({\text{x}}_{i}\right)\right)/2$

11:   With ${\text{Y}}_{n_{i}}$,

12:    $\ell_{i}\left({\hat{g}}_{\text{z},i}\right)\leftarrow \sum_{t=n_{i-1}+1}^{n_{i}}[y_{t}^{2}e^{-{\hat{g}}_{\text{z},i}\left({\text{x}}_{t}\right)}+{\hat{g}}_{\text{z},i}\left({\text{x}}_{t}\right)]+\delta {\left\|{\hat{\text{w}}}_{\text{z},i}\right\|}^{2}/2$

13:  **end for**

14:  $\ell^{*}\left({\hat{g}}_{\text{z}}\right)\leftarrow \sum_{i=2}^{k}\ell_{i}\left({\hat{g}}_{\text{z},i}\right)$

15: **end for**

16: ${\text{z}}^{*}\leftarrow {\text{argmin}}_{\text{z}}\ell^{*}\left({\hat{g}}_{\text{z}}\right)$

17: ${\hat{g}}_{{\text{z}}^{*}}\leftarrow {\text{argmin}}_{\left({\text{w}}_{{\text{z}}^{*}},b_{{\text{z}}^{*}}\right)}\left({\text{w}}_{{\text{z}}^{*}}^{T}{\text{w}}_{{\text{z}}^{*}}+b_{{\text{z}}^{*}}^{2}\right)/2+C\sum_{i=1}^{n}L\left(y_{i}-g_{{\text{z}}^{*}}\left({\text{x}}_{i}\right)\right)/2$

   **return** ${\hat{g}}_{{\text{z}}^{*}}$

### 3.2 Constructing OCC-based control chart

In this study, OCC-based control chart refers to the control chart that exploits the properties of *ρ* ∈ (0, 1) in (11) mentioned in Section 2.2 to prescribe a desired level of the control limit. Given model residuals ${\hat{\epsilon }}_{t}=y_{t}/{\hat{\sigma }}_{t}$ and a set of tuning parameters (*ρ*, *κ*), where ${\hat{\sigma }}_{t}$ is estimated by using any suitable estimation method, we fit SVDD with the Gaussian kernel presented in (16) to the squared residuals ${\hat{\epsilon }}_{t}^{2}$ obtained from the training time series, and regard *R*^2^ as the control limit. Moreover, when a new time series *y*_*n*+*k*_ (*k* ≥ 1) is observed, we obtain its squared residuals ${\hat{\epsilon }}_{n+k}^{2}$ and then measure the distance $D{\left({\hat{\epsilon }}_{n+k}^{2}\right)}^{2}$ defined in (10) against the origin of hypersphere for each *k*, and declare the process out-of-control if it exceeds *R*^2^.

The control limit is determined by leveraging two tuning parameters, namely, the inclusion rate *ρ* and the kernel tuning parameter *κ*, which settle the in-control average run length (ARL) to a desired level. To find the optimal tuning parameters, we use a method that hybridizes the standard grid searching method and quasi-Newton methods, such as the limited-memory BFGS algorithm [50]. This is because solely relying on the latter method or other first-order optimization methods may render tuning parameters not to converge when the provided length of the training time series is insufficient.

Although the general procedure to construct the OCC-based control chart in this study resembles those of [47], our OCC-based control chart is designed to preserve the original structure of the given time series compared to the latter. In implementation, OCC charts of [47] must sequentially perform the Yeo-Johnson transformation and the moving-average smoothing to the residuals to alleviate the instability caused by the inaccurate estimation of the model parameters. Despite these efforts, OCC charts had a relatively inferior performance in terms of ARL_1_ compared to other charts, such as CUSUM or EWMA charts, when monitoring the conditional volatility using squared residuals, see Section 4 of [47]. Furthermore, as our simulation study indicates, smoothing residuals undermines the overall detection ability when the underlying model is contaminated with noises, see Section 4 of this paper for more detail. We actually suspect that such posterior manipulations on the residuals ignited this phenomenon by contaminating the information that the time series originally contained. However, as our OCC-based chart directly utilizes the squared residuals without any alterations, it not only facilitates the model residuals to retain the original structure, but also enhances the detection ability, as witnessed in the simulation results in Section 4.

The gist of constructing the OCC-based control chart, given a time series and some adequate models, is summarized in Algorithms 2 and 3 below. Specifically, Algorithm 2 describes the procedure of implementing SVDD for building an OCC-based control chart, given two tuning parameters *ρ* and *κ*. Meanwhile, Algorithm 3 provides an instruction to formulate OCC-based control charts using SVDD.

**Remark 1** *Under the circumstance in which obtaining copies of in-control time series is infeasible, we can empirically optimize tuning parameters by performing a wild-bootstrap method as specified below*:

1. *Estimate* $\hat{g}$ *from the training time series y*_1_, …, *y*_*m*_, *then recursively compute* ${\hat{\sigma }}_{t}^{2}$ *using* (13). *When parametric models are employed, we instead estimate the model parameters*;
2. *Generate an iid sequence of standard normal random variables of length n, namely*, $\epsilon_{t}^{b}$, *t* = 1, …, *n*, *b* = 1, …, *B, and construct a sequence of bootstrap samples* $y_{t}^{b}={\hat{\sigma }}_{t}\epsilon_{t}^{b}$;
3. *Obtain the residuals of bootstrap samples by computing* ${\hat{\epsilon }}_{t}^{b}=y_{t}^{b}/{\hat{\sigma }}_{t}^{b}$ , *where* ${\hat{\sigma }}_{t}^{b2}=\hat{g}\left(y_{t-1}^{b2},{\tilde{\sigma }}_{t-1}^{b2}\right)$;
4. *Based on* ${\hat{\epsilon }}_{t}^{b2}$ , *fit SVDD and adjust its tuning parameters accordingly to obtain the control limit R*^2^ *that sets the in-control ARL to the desired level*.

*The validity of this method is discussed in the simulation experiments in Section 4. Also, this method is adopted when analyzing financial time series in Section 5, where only a small amount of training sample is available*.

**Algorithm 2** SVDD_**b**_ for OCC-based control charts

 **Input**: A sequence of residuals ${\left\{{\hat{\epsilon }}_{t}\right\}}_{t=1}^{n}$, a kernel *K*_2_, a vector of tuning parameters **b** = (*C*_2_, *κ*)

 **Output**: Dual variables ***α*** = (*α*_1_, ⋯, *α*_*n*_), a control limit candidate *R*^2^

1: $S\leftarrow \{0\leq \alpha_{i}\leq C_{2},\;\sum_{i}\alpha_{i}=1\}$

2: Given ${\left\{{\hat{\epsilon }}_{t}\right\}}_{t=1}^{n}$ and **b** ≔ (*ρ*, *κ*),

3:  ${\hat{\alpha }}^{\text{b}}≔\left({\hat{\alpha }}_{1}^{\text{b}},\cdots ,{\hat{\alpha }}_{n}^{\text{b}}\right)\leftarrow {\text{argmax}}_{\alpha_{i}\in S}\sum_{i=1}^{n}\alpha_{i}K_{2}\left({\hat{\epsilon }}_{i}^{2},{\hat{\epsilon }}_{i}^{2}\right)-\sum_{i,j=1}^{n}\alpha_{i}\alpha_{j}K_{2}\left({\hat{\epsilon }}_{i}^{2},{\hat{\epsilon }}_{j}^{2}\right)$

4:  $R_{\text{b}}^{2}\leftarrow K_{2}\left({\hat{\epsilon }}_{sv}^{2},{\hat{\epsilon }}_{sv}^{2}\right)-2\sum_{i=1}^{n}{\hat{\alpha }}_{i}^{\text{b}}K_{2}\left({\hat{\epsilon }}_{sv}^{2},{\hat{\epsilon }}_{i}^{2}\right)+\sum_{i,j=1}^{n}{\hat{\alpha }}_{i}^{\text{b}}{\hat{\alpha }}_{j}^{\text{b}}K_{2}\left({\hat{\epsilon }}_{i}^{2},{\hat{\epsilon }}_{j}^{2}\right)$ (${\hat{\epsilon }}_{sv}^{2}$: a support vector)

   **return** ${\hat{\alpha }}^{\text{b}}$ , $R_{\text{b}}^{2}$

**Algorithm 3** OCC-based control chart for nonlinear GARCH models

 **Input**: A training set ${\left\{y_{t}\right\}}_{t=1}^{n}$, an estimated model $\hat{g}$,

    *N* streams of in-control time series {*z*_*tj*_}_*t*≥1_ (*j* = 1, …, *N*),

    a kernel *K*_2_, *s* ≥ 1, a desired level of in-control ARL c*, a tolerance *ϵ*_*tol*_ > 0,

    a collection of tuning parameters for SVDD B

 **Output**: A control limit *R*^2^

1: **for** 1 ≤ *t* ≤ *n* **do**

2:  ${\tilde{\sigma }}_{t}^{2}\leftarrow \sum_{k=1}^{s}y_{t-k+1}^{2}/s$; $\text{log}\;{\hat{\sigma }}_{t}^{2}\leftarrow \hat{g}\left(y_{t-1}^{2},{\tilde{\sigma }}_{t-1}^{2}\right)$

3:  ${\hat{\epsilon }}_{t}\leftarrow y_{t}/{\hat{\sigma }}_{t}$

4: **end for**

5: $S\leftarrow \{0\leq \alpha_{i}\leq C,\;\sum_{i=1}^{n}\alpha_{i}=1\}$

6: With ${\left\{{\hat{\epsilon }}_{t}\right\}}_{t=1}^{n}$,

7: **for** each pair of b∈B **do**

8:  $\left({\hat{\alpha }}^{\text{b}},R_{\text{b}}^{2}\right)\leftarrow {\text{SVDD}}_{\text{b}}\left(\left\{{\hat{\epsilon }}_{t}\right\},K_{2},\text{b}\right)$

9:  With ${\left\{z_{tj}\right\}}_{t=1}^{n}$,

10:  **for** 1 ≤ *j* ≤ *N* **do**

11:   **for** *t* ≥ 1 **do**

12:    ${\tilde{\sigma }}_{t,j}^{2}\leftarrow \sum_{k=1}^{s}z_{t-k+1,j}^{2}/s$; $\text{log}{\hat{\sigma }}_{t,j}^{2}\leftarrow \hat{g}\left(z_{t-1,j}^{2},{\tilde{\sigma }}_{t-1,j}^{2}\right)$

13:    ${\hat{\epsilon }}_{t,j}\leftarrow z_{t,j}/{\hat{\sigma }}_{t,j}$

14:    $D^{2}\left(z_{tj}\right)\leftarrow K_{2}\left({\hat{\epsilon }}_{t,j}^{2},{\hat{\epsilon }}_{t,j}^{2}\right)-2\sum_{i=1}^{n}{\hat{\alpha }}_{i}^{\text{b}}K_{2}\left({\hat{\epsilon }}_{t,j}^{2},{\hat{\epsilon }}_{i}^{2}\right)+\sum_{i,j=1}^{n}{\hat{\alpha }}_{i}^{\text{b}}{\hat{\alpha }}_{j}^{\text{b}}K_{2}\left({\hat{\epsilon }}_{i}^{2},{\hat{\epsilon }}_{j}^{2}\right)$

15:    **if** $D^{2}\left(z_{tj}\right)\geq R_{\text{b}}^{2}$ **then**

16:     $RL_{j,R_{\text{b}}^{2}}\leftarrow {\text{inf}}_{t\geq 1}\left\{D^{2}\left(z_{tj}\right)\geq R_{\text{b}}^{2}\right\}$; **break**

17:    **end if**

18:   **end for**

19:  **end for**

20:  $ARL_{0,R_{\text{b}}^{2}}\leftarrow \sum_{j=1}^{N}RL_{j,R_{\text{b}}^{2}}/N$

21: **end for**

22: ${\text{b}}^{*}\leftarrow (\text{b}\in B\;\text{such}\;\text{that}\;|ARL_{0,R_{\text{b}}^{2}}-c^{*}\left|<\epsilon_{tol}\right)$

23: $\left({\hat{\alpha }}^{{\text{b}}^{*}},R_{{\text{b}}^{*}}^{2}\right)\leftarrow {\text{SVDD}}_{\text{b}}\left(\left\{{\hat{\epsilon }}_{t}\right\},K_{2},{\text{b}}^{*}\right)$

    **return** $R_{{\text{b}}^{*}}^{2}$

## 4 Simulation results

This section assesses the performance of control charts that utilizes HSVR residuals in various nonlinear GARCH models. The first subsection describes the settings of the experiment in more detail, and the subsequent subsection reports the performance measured in terms of the average run length (ARL).

### 4.1 Specifications of the experiment

For the experiment, we consider the cases where the underlying models are GJR-GARCH(*p*, *q*) [51] and log-GARCH(*p*, *q*) specified below:

$$\begin{array}{cc} •\;\text{GJR-GARCH}\left(p,q\right):\;\;\;y_{t} & =\sigma_{t}\epsilon_{t},\\ \sigma_{t}^{2} & =\omega +\sum_{i=1}^{p}\left(\alpha_{1,i}{y_{t-i}^{+}}^{2}+\alpha_{2,i}{y_{t-i}^{-}}^{2}\right)+\sum_{j=1}^{q}\beta_{j}\sigma_{t-j}^{2},\\ y_{t}^{+} & =\text{max}\left(y_{t},\;0\right),\;y_{t}^{-}=-\text{min}\left(y_{t},\;0\right),\\ \alpha_{1,i} & \geq 0,\alpha_{2,i}\geq 0,\beta_{j}\geq 0;\\ •\;\text{log-GARCH}\left(p,q\right):\;\;\;y_{t} & =\sigma_{t}\epsilon_{t},\\ \text{log}\;\sigma_{t}^{2} & =\omega +\sum_{i=1}^{p}\alpha_{i}\;\text{log}\;y_{t-i}^{2}+\sum_{j=1}^{q}\beta_{j}\;\text{log}\;\sigma_{t-j}^{2}, \end{array}$$

where *ω* ≥ 0 and {*ϵ*_*t*_} is an iid random process. Here, the log-GARCH(1,1) model is a variation of the exponential GARCH model (i.e., EGARCH(*p*, *q*); see [42, 52]), which has a high degree of nonlinearity. In this experiment, we set *p* = *q* = 1 and set (*ω*, *α*_1_, *α*_2_, *β*) = (0.3, 0.1, 0.5, 0.3) for the GJR-GARCH model, and (*ω*, *α*, *β*) = (0.3, 0.3, 0.3) for the log-GARCH model.

Moreover, to further reflect the circumstance where the underlying model is highly volatile and unstable, we additionally consider the cases where the initial distribution of *ϵ*_*t*_ in the training time series is specified as follows:

- **Case 1**. *ϵ*_*t*_ ∼ *N*(0, 1), the standard normal distribution;
- **Case 2**. *ϵ*_*t*_ ∼ *t*(5), *t*-distribution with 5 degrees of freedom;
- **Case 3**. *ϵ*_*t*_ ∼ *Z*_*k*_ = 0.9*X* + 0.1*Y*, *X* ∼ *N*(0, 1), *Y* ∼ *N*(0, *k*^2^).
- **Case 4**. *ϵ*_*t*_ ∼ *Z*_*l*, *m*_ = 0.9*X* + 0.1*W*, *X* ∼ *N*(0, 1), *W* ∼ *N*(*l*, *m*^2^).

The latter three distributions symbolize the scenarios in which the observed time series is either inherently heavy-tailed or occasionally unstable. In particular, the latter two distributions are variants of a normal mixture distribution that represents the underlying model being highly volatile due to the innovational outliers. Specifically, *Z*_*k*_ and *Z*_*l*, *m*_ respectively denote that the underlying structure of time series is systematically unstable and asymmetric, which are some traits that can be witnessed in various financial time series. In this study, we consider the case of *k* = 3 and (*l*, *m*) = (1, 2).

In addition to considering innovational outliers, we also contaminate the observed time series with additive outliers in some experiments. Namely, we consider the case where we observe $y_{t}^{\prime }=y_{t}+\eta_{t}$ rather than directly observing *y*_*t*_, where *η*_*t*_ follows some prescribed distribution function. For this experiment, we respectively set *η*_*t*_ analogous to Cases 1 and 4 for GJR-GARCH model, and to Cases 3 and 4 for log-GARCH model when additive outliers are taken into consideration in the model.

When comparing the performance, we evaluate ARLs of three control charts, namely, the proposed OCC-based control chart in Section 3, CUSUM chart, and EWMA chart, that uses squared residuals. Here, CUSUM chart refers to the control chart that utilizes

$$\begin{array}{c} C_{t}^{+}=\text{max}\left(0,{\hat{\epsilon }}_{t}^{2}-\mu -K+C_{t-1}^{+}\right),\\ C_{t}^{-}=\text{max}\left(0,\mu -{\hat{\epsilon }}_{t}^{2}-K+C_{t-1}^{-}\right), \end{array}$$

where *K* = *kσ*, *H* = *hσ*, *σ* denotes the standard deviation of squared residuals ${\hat{\epsilon }}_{t}^{2}$ obtained from the training time series, and *μ* denotes the mean of ${\hat{\epsilon }}_{t}^{2}$. On the other hand, EWMA chart denotes the control chart that uses

$$\begin{array}{c} Z_{t}=\lambda {\hat{\epsilon }}_{t}^{2}+\left(1-\lambda \right)Z_{t-1}, \end{array}$$

with the upper and lower control limits computed as

$$\begin{array}{c} UCL=\mu +L_{e}\sigma \sqrt{\frac{\lambda }{2-\lambda }},\;\;LCL=\mu +L_{e}\sigma \sqrt{\frac{\lambda }{2-\lambda }}, \end{array}$$

where λ ∈ (0, 1) is a smoothing parameter, and *Z*_0_ is defined to be the sample mean of ${\hat{\epsilon }}_{t}^{2}$. Notice that the control limit of CUSUM and EWMA charts are respectively controlled by tuning *k* ≥ 0, *h* ≥ 0, or *L*_*e*_ ≥ 0, to dictate control charts to achieve the desired level of ARL_0_. For an overview of these control charts, we refer to [53–55].

Moreover, as we regard the underlying structure of the time series is unknown, we compare the detection ability of control charts using HSVR-GARCH against those that utilizes the standard GARCH(1,1) model specified below:

$$\begin{array}{cc} y_{t} & =\sigma_{t}\epsilon_{t},\\ \sigma_{t}^{2} & =\omega +\alpha y_{t-1}^{2}+\beta \sigma_{t-1}^{2}, \end{array}$$ (17)

where *ω*, *α*, and *β* are all nonnegative, and *α* + *β* < 1 is fulfilled to ensure the stationarity. Notice that fitting GARCH(1,1) models to some other conditionally heteroscedastic time series is customarily accepted in the presence the model uncertainty, see [56].

The general procedure of the experiment is summarized as follows. We first generate a time series of length *n* for training either the HSVR-GARCH model stated in Section 3 or the standard GARCH(1,1) model to obtain the residuals. We subsequently use squared residuals ${\hat{\epsilon }}_{t}^{2}$ to formulate the control chart, as they are solely designed to target the change of variance. We then sequentially generate 1,000 independent streams of time series without any structural changes in order to find adequate tuning parameters that fix ARL_0_ to the desired level, for example, the frequently used 370, though this number must be reduced significantly in a practical situation, as mentioned later in the real data analysis. To evaluate the detection ability, we generate another 1,000 independent streams of time series that includes a structural change, then apply the control charts to obtain ARL_1_.

We set the length of the training time series as *n* = 2, 000 and examine ARL_1_ of control charts when some parameters, innovational distribution of *ϵ*_*t*_, or the magnitude of the additive noise experience a change, respectively. Particularly, we specify the way in which the structural change occurs in Tables 1–8 below, alongside with the respective tuning parameters for each used control charts. When fitting HSVR-GARCH to time series, we initially fix *δ* = 0.1 and the number of chunks *k* to 5, then obtain other tuning parameters by employing the particle swarm optimization method.

**Table 1:** A comparison of control charts using ${\hat{\epsilon }}_{t}^{2}$ and ${\tilde{\epsilon }}_{t}^{2}$ when the underlying model is GJR-GARCH(1,1) with the specified parameters without any additive outliers. Results of the first two columns directly compares our OCC-based control chart with that of [47].

			(*ω*, *α*_1_, *α*_2_, *β*) = (0.3, 0.1, 0.5, 0.3)					
			OCC		CUSUM		EWMA	
			${\hat{\epsilon }}_{t}^{2}$	${\tilde{\epsilon }}_{t}^{2}$	${\hat{\epsilon }}_{t}^{2}$	${\tilde{\epsilon }}_{t}^{2}$	${\hat{\epsilon }}_{t}^{2}$	${\tilde{\epsilon }}_{t}^{2}$
			*ρ* = 0.9975	*ρ* = 0.997	*k* = 0.5	*k* = 0.5	λ = 0.2	λ = 0.2
			*κ* = 25	*κ* = 5000	*h* = 4.9	*h* = 5.6	*L*_*e*_ = 3.2	*L*_*e*_ = 3.5
*η*_*t*_ ∼ *Z*_1,2_	ARL_0_		372.875	370.113	372.114	371.928	370.636	373.206
	ARL_1_	*ω* → 0.5	183.976	271.464	167.290	189.777	170.625	188.899
		*ω* → 0.7	118.586	191.426	110.352	120.795	112.920	120.431
		*ω* → 1	70.571	119.32	60.424	71.326	62.238	70.008
		*α*_1_ → 0.6	68.967	93.691	65.222	72.016	66.691	70.707
		*α*_1_ → 0.9	37.599	44.386	33.422	33.044	34.224	33.564
		*α*_2_ → 1	120.675	151.936	111.021	111.82	112.611	109.393
		*α*_2_ → 1.3	76.464	87.927	72.477	74.016	72.445	72.465
		*β* → 0.6	62.567	92.202	56.249	60.833	56.434	59.996
		*β* → 0.65	34.761	54.836	32.142	34.445	32.750	34.7
		*η*_*t*_ ∼ *Z*_3_	48.602	53.879	44.631	40.601	45.632	41.592
		*η*_*t*_ ∼ *Z*_5_	28.413	34.37	26.421	26.646	26.814	26.753

**Table 2:** ARLs of control charts when the underlying model is GJR-GARCH(1,1) with the specified parameters, where no additive outliers are present. “HSVR” and “GARCH” denotes that the chart is constructed using residuals obtained from fitting HSVR and GARCH(1,1) models, respectively.

			(*ω*, *α*_1_, *α*_2_, *β*) = (0.3, 0.1, 0.5, 0.3)					
			OCC		CUSUM		EWMA	
			HSVR	GARCH	HSVR	GARCH	HSVR	GARCH
			*ρ* = 0.9985	*ρ* = 0.9968	*k* = 0.5	*k* = 0.5	λ = 0.2	λ = 0.2
			*κ* = 7.5	*κ* = 7.5	*h* = 8.8	*h* = 7.299	*L*_*e*_ = 4.5	*L*_*e*_ = 4.1
*η*_*t*_ ∼ *N*(0, 1)	ARL_0_		370.446	371.038	370.840	372.716	369.723	363.220
	ARL_1_	*ω* → 0.5	136.302	82.962	98.007	96.657	107.910	99.607
		*ω* → 0.7	64.473	45.342	44.501	40.750	50.988	44.909
		*ω* → 1	33.566	30.956	22.301	22.027	26.330	25.771
		*α*_1_ → 0.6	80.205	83.976	58.439	73.192	61.498	78.089
		*α*_1_ → 0.9	38.850	48.394	31.401	36.220	33.019	39.200
		*α*_2_ → 1	81.644	75.719	74.051	74.645	74.825	76.242
		*α*_2_ → 1.3	47.137	47.392	45.954	43.582	47.357	45.001
		*β* → 0.6	41.029	37.422	31.522	30.047	34.968	33.366
		*β* → 0.65	25.950	25.205	20.218	20.398	22.162	22.346
		*η*_*t*_ ∼ *Z*_3_	39.195	30.185	44.279	31.696	46.628	32.815
		*η*_*t*_ ∼ *Z*_5_	20.587	17.827	23.063	18.719	23.255	19.095
			*ρ* = 0.9945	*ρ* = 0.9968	*k* = 0.5	*k* = 0.5	λ = 0.2	λ = 0.2
			*κ* = 5000	*κ* = 37.5	*h* = 1.9	*h* = 7	*L*_*e*_ = 1.5	*L*_*e*_ = 4.307
*η*_*t*_ ∼ *t*(5)	ARL_0_		365.632	371.841	372.419	370.282	366.592	370.255
	ARL_1_	*ω* → 0.5	193.490	2268.776	181.007	202.652	176.334	204.420
		*ω* → 0.7	141.582	1707.191	129.491	136.518	122.790	140.515
		*ω* → 1	86.650	1307.601	75.821	99.736	71.701	101.836
		*α*_1_ → 0.6	73.675	1883.895	66.336	126.011	63.233	138.586
		*α*_1_ → 0.9	34.429	1150.821	31.245	62.582	30.287	71.039
		*α*_2_ → 1	97.308	1728.632	90.019	129.821	87.637	137.864
		*α*_2_ → 1.3	54.701	1053.547	51.760	75.619	51.519	82.713
		*β* → 0.6	56.391	1045.853	51.476	79.663	49.137	84.799
		*β* → 0.65	27.691	631.713	25.872	43.929	24.575	48.187
		*η*_*t*_ ∼ *Z*_5_	277.600	2473.391	275.660	170.155	273.325	182.807
		*η*_*t*_ ∼ *Z*_7_	67.277	268.222	65.450	41.018	67.617	44.037
			*ρ* = 0.994	*ρ* = 0.9955	*k* = 0.5	*k* = 0.5	λ = 0.2	λ = 0.2
			*κ* = 50	*κ* = 25	*h* = 0.9	*h* = 9	*L*_*e*_ = 0.9	*L*_*e*_ = 5.3
*η*_*t*_ ∼ *Z*_3_	ARL_0_		370.478	368.596	371.991	371.558	371.945	367.150
	ARL_1_	*ω* → 0.5	190.921	1194.113	193.944	177.293	190.784	178.126
		*ω* → 0.7	125.069	701.277	130.086	126.626	124.039	124.331
		*ω* → 1	80.851	402.286	83.458	85.364	83.16	85.626
		*α*_1_ → 0.6	39.652	300.517	39.677	60.723	36.887	68.728
		*α*_1_ → 0.9	24.444	167.595	24.514	38.362	23.911	41.766
		*α*_2_ → 1	106.32	576.124	106.035	145.578	101.887	150.816
		*α*_2_ → 1.3	62.031	320.945	63.465	96.085	61.004	99.925
		*β* → 0.6	52.142	273.508	52.51	66.265	51.249	68.765
		*β* → 0.65	29.213	141.928	29.888	39.22	29.23	44.206
		*η*_*t*_ ∼ *Z*_3_	75.858	170.707	79.507	56.27	81.725	58.146
		*η*_*t*_ ∼ *Z*_5_	40.28	57.671	40.611	28.645	42.022	29.539

**Table 3:** ARLs of control charts when the underlying model is GJR-GARCH(1,1) with the specified parameters, where the dataset is contaminated with a *N*(0, 1)-distributed noise.

			(*ω*, *α*_1_, *α*_2_, *β*) = (0.3, 0.1, 0.5, 0.3)					
			OCC		CUSUM		EWMA	
			HSVR	GARCH	HSVR	GARCH	HSVR	GARCH
			*ρ* = 0.995	*ρ* = 0.9978	*k* = 0.5	*k* = 0.5	λ = 0.2	λ = 0.2
			*κ* = 100	*κ* = 25	*h* = 0.4	*h* = 7.1	*L*_*e*_ = 0.6	*L*_*e*_ = 4.2
*η*_*t*_ ∼ *t*(5)	ARL_0_		366.039	351.275	367.542	367.743	373.310	373.955
	ARL_1_	*ω* → 0.5	187.383	1172.510	190.715	212.714	201.027	207.763
		*ω* → 0.7	129.060	818.789	122.151	124.821	122.953	126.681
		*ω* → 1	88.614	524.571	83.888	83.225	84.588	84.663
		*α*_1_ → 0.6	71.568	413.161	69.759	75.936	67.574	78.377
		*α*_1_ → 0.9	36.648	173.231	33.402	36.351	32.178	39.136
		*α*_2_ → 1	92.575	450.002	90.976	98.209	90.290	101.190
		*α*_2_ → 1.3	60.754	236.748	55.311	61.230	55.440	63.298
		*β* → 0.6	45.867	205.138	45.540	44.614	43.789	47.363
		*β* → 0.65	26.158	108.279	24.651	24.881	23.939	27.051
		*η*_*t*_ ∼ *Z*_3_	61.888	353.855	59.004	49.493	60.776	49.470
		*η*_*t*_ ∼ *Z*_5_	33.135	121.309	31.756	28.218	32.209	28.451
			*ρ* = 0.9958	*ρ* = 0.9955	*k* = 0.5	*k* = 0.5	λ = 0.2	λ = 0.2
			*κ* = 750	*κ* = 100	*h* = 1.5	*h* = 7.9	*L*_*e*_ = 1.3	*L*_*e*_ = 4.601
*η*_*t*_ ∼ *Z*_3_	ARL_0_		372.261	367.727	370.766	369.987	372.554	369.355
	ARL_1_	*ω* → 0.5	208.093	3737.450	193.983	200.160	200.834	202.664
		*ω* → 0.7	138.967	3178.429	125.983	130.208	129.208	128.901
		*ω* → 1	90.997	2249.618	79.570	82.026	80.000	80.510
		*α*_1_ → 0.6	78.009	1568.318	70.878	85.813	68.836	90.982
		*α*_1_ → 0.9	42.836	497.984	40.095	47.034	39.090	50.564
		*α*_2_ → 1	110.517	1441.263	101.141	120.378	101.180	124.457
		*α*_2_ → 1.3	65.314	689.723	61.024	73.852	60.852	75.583
		*β* → 0.6	58.779	554.463	54.576	55.839	52.257	59.324
		*β* → 0.65	32.553	252.352	29.467	31.752	28.966	34.648
		*η*_*t*_ ∼ *Z*_5_	87.480	637.316	77.121	69.162	83.474	70.064
		*η*_*t*_ ∼ *Z*_7_	45.266	165.279	40.219	35.362	42.512	36.461
			*ρ* = 0.9985	*ρ* = 0.9958	*k* = 0.5	*k* = 0.5	λ = 0.2	λ = 0.2
			*κ* = 7.5	*κ* = 3.75	*h* = 6.9	*h* = 7.3	*L*_*e*_ = 4.2	*L*_*e*_ = 4.3
*η*_*t*_ ∼ *Z*_1,2_	ARL_0_		372.108	370.961	372.485	366.699	372.548	370.087
	ARL_1_	*ω* → 0.5	152.072	2969.550	140.145	137.474	136.998	139.763
		*ω* → 0.7	90.360	2007.426	83.533	75.755	83.930	81.874
		*ω* → 1	56.452	1016.499	46.359	42.687	46.886	48.635
		*α*_1_ → 0.6	63.088	643.952	55.779	55.929	56.080	61.372
		*α*_1_ → 0.9	34.043	199.762	30.138	30.813	30.593	33.045
		*α*_2_ → 1	109.689	1002.529	98.159	98.758	99.081	104.814
		*α*_2_ → 1.3	64.894	453.702	57.353	57.033	57.554	61.542
		*β* → 0.6	52.953	506.198	45.744	43.967	45.760	47.997
		*β* → 0.65	30.900	235.429	25.621	24.750	26.156	28.533
		*η*_*t*_ ∼ *Z*_3_	40.212	133.847	37.112	36.618	37.471	38.933
		*η*_*t*_ ∼ *Z*_5_	23.467	54.500	22.329	22.341	22.672	23.105

**Table 4:** ARLs of the OCC-based, CUSUM, and EWMA control charts when the underlying model is GJR-GARCH(1,1) with the specified parameters, where the dataset is contaminated with a *Z*_1,2_-distributed noise.

			(*ω*, *α*_1_, *α*_2_, *β*) = (0.3, 0.1, 0.5, 0.3)					
			OCC		CUSUM		EWMA	
			HSVR	GARCH	HSVR	GARCH	HSVR	GARCH
			*ρ* = 0.9958	*ρ* = 0.9953	*k* = 0.5	*k* = 0.5	λ = 0.2	λ = 0.2
			*κ* = 25	*κ* = 25	*h* = 7.7	*h* = 6.7	*L*_*e*_ = 4.8	*L*_*e*_ = 4.1
*η*_*t*_ ∼ *t*(5)	ARL_0_		369.259	366.421	373.848	373.427	374.161	367.189
	ARL_1_	*ω* → 0.5	212.459	3064.010	207.885	221.242	211.338	213.100
		*ω* → 0.7	134.997	2375.100	132.008	132.267	128.898	131.635
		*ω* → 1	85.195	1769.106	83.863	84.521	83.928	83.600
		*α*_1_ → 0.6	76.536	1062.019	73.656	77.308	72.194	80.790
		*α*_1_ → 0.9	36.524	380.421	35.878	37.295	34.208	38.695
		*α*_2_ → 1	96.631	1067.994	91.292	97.154	90.662	96.163
		*α*_2_ → 1.3	59.622	500.959	58.948	60.163	58.008	60.738
		*β* → 0.6	51.633	545.070	49.031	48.726	48.858	50.230
		*β* → 0.65	28.707	234.293	27.012	25.502	25.917	27.504
		*η*_*t*_ ∼ *Z*_3_	73.240	387.974	68.137	63.971	69.843	64.126
		*η*_*t*_ ∼ *Z*_5_	39.889	132.528	37.560	34.897	39.196	35.899
			*ρ* = 0.995	*ρ* = 0.9965	*k* = 0.5	*k* = 0.5	λ = 0.2	λ = 0.2
			*κ* = 37.5	*κ* = 10	*h* = 1.3	*h* = 7.6	*L*_*e*_ = 1.2	*L*_*e*_ = 4.5
*η*_*t*_ ∼ *Z*_3_	ARL_0_		369.918	370.792	365.555	371.178	373.109	370.929
	ARL_1_	*ω* → 0.5	192.728	1332.195	197.218	206.531	211.703	204.694
		*ω* → 0.7	138.662	846.585	133.014	139.207	145.337	135.719
		*ω* → 1	92.323	461.804	80.852	88.533	87.222	90.486
		*α*_1_ → 0.6	81.806	465.786	79.352	101.892	76.077	106.310
		*α*_1_ → 0.9	42.751	192.788	38.548	51.450	38.349	55.009
		*α*_2_ → 1	105.621	493.525	95.996	118.622	98.106	125.158
		*α*_2_ → 1.3	65.761	277.020	59.581	74.145	59.832	78.926
		*β* → 0.6	57.287	214.887	53.127	59.969	53.170	62.837
		*β* → 0.65	31.066	105.212	26.216	31.656	25.560	33.604
		*η*_*t*_ ∼ *Z*_5_	92.910	237.212	83.968	77.395	90.518	78.658
		*η*_*t*_ ∼ *Z*_7_	49.775	84.231	40.011	37.193	45.376	38.220
			*ρ* = 0.9975	*ρ* = 0.9945	*k* = 0.5	*k* = 0.5	λ = 0.2	λ = 0.2
			*κ* = 25	*κ* = 7.5	*h* = 4.9	*h* = 7.296	*L*_*e*_ = 3.2	*L*_*e*_ = 4.3
*η*_*t*_ ∼ *Z*_1,2_	ARL_0_		372.875	368.229	372.114	368.880	370.636	374.419
	ARL_1_	*ω* → 0.5	183.976	4122.703	167.290	178.584	170.625	181.809
		*ω* → 0.7	118.586	3573.780	110.352	116.682	112.920	116.029
		*ω* → 1	70.571	2536.990	60.424	63.125	62.238	66.798
		*α*_1_ → 0.6	68.967	1566.241	65.222	67.235	66.691	72.853
		*α*_1_ → 0.9	37.599	437.241	33.422	36.124	34.224	38.631
		*α*_2_ → 1	120.675	2052.610	111.021	117.285	112.611	119.310
		*α*_2_ → 1.3	76.464	903.953	72.477	74.618	72.445	78.730
		*β* → 0.6	62.567	1362.131	56.249	57.197	56.434	61.778
		*β* → 0.65	34.761	573.701	32.142	32.134	32.750	35.619
		*η*_*t*_ ∼ *Z*_3_	48.602	248.021	44.631	43.510	45.632	45.849
		*η*_*t*_ ∼ *Z*_5_	28.413	86.653	26.421	26.159	26.814	27.189

**Table 5:** ARLs of control charts when the underlying model is log-GARCH(1,1) with the specified parameters, where no additive outliers are present.

			(*ω*, *α*, *β*) = (0.3, 0.3, 0.3)					
			OCC		CUSUM		EWMA	
			HSVR	GARCH	HSVR	GARCH	HSVR	GARCH
			*ρ* = 0.994	*ρ* = 0.9933	*k* = 0.5	*k* = 0.5	λ = 0.2	λ = 0.2
			*κ* = 50	*κ* = 2.5	*h* = 11.6	*h* = 6	*L*_*e*_ = 5.8	*L*_*e*_ = 3.6
*η*_*t*_ ∼ *N*(0, 1)	ARL_0_		370.074	363.373	367.762	364.031	377.254	376.14
	ARL_1_	*ω* → 0.5	96.953	204.157	81.435	120.385	90.099	124.069
		*ω* → 0.7	34.668	107.347	30.75	50.464	34.121	52.297
		*ω* → 1	11.667	47.632	11.513	18.483	12.187	21.994
		*α* → 0.5	334.772	1288.594	327.375	644.089	327.483	895.936
		*α* → −0.3	31.771	12.43	41.219	11.712	46.881	12.553
		*α* → −0.5	14.425	8.71	18.212	8.233	18.939	8.956
		*β* → 0.6	224.202	534.148	213.409	167.335	221.026	342.104
		*β* → 0.65	174.576	438.568	162.305	305.35	169.739	314.971
		*η*_*t*_ ∼ *Z*_3_	42.003	81.577	47.18	33.106	47.299	34.788
		*η*_*t*_ ∼ *Z*_5_	21.832	29.845	25.289	18.772	24.911	19.49
			*ρ* = 0.9935	*ρ* = 0.9958	*k* = 0.5	*k* = 0.5	λ = 0.2	λ = 0.2
			*κ* = 1000	*κ* = 50	*h* = 8	*h* = 6.4	*L*_*e*_ = 4.8	*L*_*e*_ = 3.9
*η*_*t*_ ∼ *t*(5)	ARL_0_		373.471	373.227	371.91	367.028	378.722	372.99
	ARL_1_	*ω* → 0.5	143.131	216.815	131.54	212.958	130.665	198.216
		*ω* → 0.7	66.418	139.217	56.219	129.513	57.249	120.69
		*ω* → 1	24.282	75.245	20.576	65.8	21.179	61.058
		*α* → 0.5	239.527	1031.768	294.276	789.714	294.491	799.746
		*α* → −0.3	137.812	37.386	105.206	30.333	504.073	30.589
		*α* → −0.5	47.723	19.177	36.698	15.611	97.081	15.991
		*β* → 0.6	242.083	355.825	210.412	350.794	213.828	347.159
		*β* → 0.65	112.565	265.819	106.112	239.076	108.782	225.168
		*η*_*t*_ ∼ *Z*_5_	55.747	279.235	59.835	42.753	59.343	43.057
		*η*_*t*_ ∼ *Z*_7_	32.321	94.725	34.038	25.179	34.253	25.703
			*ρ* = 0.9958	*ρ* = 0.9948	*k* = 0.5	*k* = 0.5	λ = 0.2	λ = 0.2
			*κ* = 2500	*κ* = 10	*h* = 9.3	*h* = 8.7	*L*_*e*_ = 5.6	*L*_*e*_ = 5.2
*η*_*t*_ ∼ *Z*_3_	ARL_0_		374.122	372.796	373.34	368.501	366.803	366.555
	ARL_1_	*ω* → 0.5	159.84	191.408	141.352	186.653	146.668	183.39
		*ω* → 0.7	76.763	122.122	64.215	113.087	68.031	111.329
		*ω* → 1	37.334	72.368	26.155	62.837	29.455	62.982
		*α* → 0.5	266.694	1207.753	323.831	919.131	337.639	938.049
		*α* → −0.3	85.041	49.093	103.123	40.903	107.275	42.49
		*α* → −0.5	38.128	25.207	43.166	21.708	44.623	22.092
		*β* → 0.6	157.764	260.313	135.793	238.829	143.327	234.512
		*β* → 0.65	135.365	224.855	123.441	205.204	127.191	204.745
		*η*_*t*_ ∼ *Z*_5_	71.951	697.218	74.986	58.871	77.334	60.605
		*η*_*t*_ ∼ *Z*_7_	39.14	139.974	39.277	31.877	40.17	31.833

**Table 6:** ARLs of control charts when the underlying model is log-GARCH(1,1) with the specified parameters, where the dataset is contaminated with a *Z*_3_-distributed noise.

			(*ω*, *α*, *β*) = (0.3, 0.3, 0.3)					
			OCC		CUSUM		EWMA	
			HSVR	GARCH	HSVR	GARCH	HSVR	GARCH
			*ρ* = 0.9928	*ρ* = 0.9933	*k* = 0.5	*k* = 0.5	λ = 0.2	λ = 0.2
			*κ* = 75	*κ* = 5000	*h* = 8.5	*h* = 7.5	*L*_*e*_ = 5.2	*L*_*e*_ = 4.4
*η*_*t*_ ∼ *t*(5)	ARL_0_		370.128	366.707	374.413	369.201	373.347	359.645
	ARL_1_	*ω* → 0.5	195.727	252.261	171.516	224.343	174.792	223.588
		*ω* → 0.7	100.674	150.211	77.762	112.777	81.467	111.672
		*ω* → 1	34.672	62.801	26.828	39.404	28.432	43.165
		*α* → 0.5	274.744	357.026	242.33	333.332	254.136	344.2
		*α* → −0.3	120.623	105.865	117.023	99.729	122.792	99.559
		*α* → −0.5	43.651	38.374	38.927	36.223	40.049	36.793
		*β* → 0.6	167.242	252.679	144.058	186.447	150.419	188.021
		*β* → 0.65	126.673	176.913	115.61	134.404	117.716	138.067
		*η*_*t*_ ∼ *Z*_3_	74.721	73.059	67.921	68.147	71.021	68.42
		*η*_*t*_ ∼ *Z*_5_	42.856	39.545	39.062	37.889	39.999	37.781
			*ρ* = 0.9965	*ρ* = 0.997	*k* = 0.5	*k* = 0.5	λ = 0.2	λ = 0.2
			*κ* = 500	*κ* = 100	*h* = 9.1	*h* = 8.2	*L*_*e*_ = 5.5	*L*_*e*_ = 4.8
*η*_*t*_ ∼ *Z*(3)	ARL_0_		363.817	367.587	370.344	369.966	366.56	367.426
	ARL_1_	*ω* → 0.5	175.376	212.805	150.738	178.199	155.972	175.154
		*ω* → 0.7	87.67	125.323	72.895	95.619	74.553	96.317
		*ω* → 1	37.511	65.477	29.784	44.175	30.656	48.421
		*α* → 0.5	302.465	336.179	278.036	298.894	287.218	305.993
		*α* → −0.3	130.983	134.087	125.107	135.345	127.509	131.403
		*α* → −0.5	49.662	53.463	45.252	51.382	47.521	50.703
		*β* → 0.6	169.802	223.312	145.505	172.816	152.308	178.675
		*β* → 0.65	136.908	178.427	121.815	140.904	126.057	146.258
		*η*_*t*_ ∼ *Z*_5_	86.207	897.751	79.221	82.824	83.63	81.829
		*η*_*t*_ ∼ *Z*_7_	47.043	221.583	44.061	45.172	45.143	46.041
			*ρ* = 0.9943	*ρ* = 0.9965	*k* = 0.5	*k* = 0.5	λ = 0.2	λ = 0.2
			*κ* = 5000	*κ* = 37.5	*h* = 9.5	*h* = 7.9	*L*_*e*_ = 5.6	*L*_*e*_ = 4.7
*η*_*t*_ ∼ *Z*_1,2_	ARL_0_		380.869	372.696	375.407	376.013	368.313	382.674
	ARL_1_	*ω* → 0.5	210.653	254.382	156.385	189.521	169.913	204.587
		*ω* → 0.7	101.193	149.116	71.532	95.989	74.5	105.054
		*ω* → 1	42.212	80.542	26.804	39.605	28.868	46.736
		*α* → 0.5	293.983	341.106	270.07	259.832	278.036	291.755
		*α* → −0.3	117.889	129.448	108.682	117.007	110.876	118.857
		*α* → −0.5	43.512	47.206	38.952	42.426	40.033	43.779
		*β* → 0.6	186.373	252.241	153.751	170.009	161.566	187.133
		*β* → 0.65	155.51	221.767	137.105	148.037	141.585	160.175
		*η*_*t*_ ∼ *Z*_5_	61.729	908.604	54.824	57.995	56.246	60.018
		*η*_*t*_ ∼ *Z*_7_	33.475	223.888	31.574	32.103	32.709	33.818

**Table 7:** ARLs of control charts when the underlying model is log-GARCH(1,1) with the specified parameters, where the dataset is contaminated with a *Z*_1,2_-distributed noise.

			(*ω*, *α*, *β*) = (0.3, 0.3, 0.3)					
			OCC		CUSUM		EWMA	
			HSVR	GARCH	HSVR	GARCH	HSVR	GARCH
			*ρ* = 0.9958	*ρ* = 0.9953	*k* = 0.5	*k* = 0.5	λ = 0.2	λ = 0.2
			*κ* = 25	*κ* = 25	*h* = 7.7	*h* = 6.7	*L*_*e*_ = 4.8	*L*_*e*_ = 4.1
*η*_*t*_ ∼ *t*(5)	ARL_0_		376.764	370.700	366.363	370.444	366.886	383.004
	ARL_1_	*ω* → 0.5	175.406	188.645	148.784	173.650	152.258	179.578
		*ω* → 0.7	78.500	106.426	62.764	81.601	65.303	86.998
		*ω* → 1	30.362	52.248	23.956	31.797	25.248	36.433
		*α* → 0.5	278.980	370.351	242.975	329.646	252.821	358.235
		*α* → −0.3	97.113	72.892	94.475	66.547	96.286	69.182
		*α* → −0.5	41.020	33.583	37.443	30.648	38.748	31.961
		*β* → 0.6	158.131	200.062	140.865	168.152	145.026	173.536
		*β* → 0.65	127.523	169.329	116.116	131.296	118.724	140.000
		*η*_*t*_ ∼ *Z*_3_	65.191	480.575	62.207	53.706	62.689	55.874
		*η*_*t*_ ∼ *Z*_5_	37.197	132.196	34.501	32.067	35.726	32.837
			*ρ* = 0.9995	*ρ* = 0.998	*k* = 0.5	*k* = 0.5	λ = 0.2	λ = 0.2
			*κ* = 2500	*κ* = 37.5	*h* = 9.3	*h* = 8.3	*L*_*e*_ = 5.6	*L*_*e*_ = 4.9
*η*_*t*_ ∼ *Z*_3_	ARL_0_		369.253	368.869	371.688	368.322	369.504	365.797
	ARL_1_	*ω* → 0.5	187.918	182.062	149.187	159.736	153.064	162.114
		*ω* → 0.7	111.718	111.298	72.547	81.715	75.275	86.105
		*ω* → 1	44.640	53.037	29.282	35.513	30.208	39.303
		*α* → 0.5	299.637	387.264	278.539	312.258	281.025	325.795
		*α* → −0.3	141.992	104.845	116.161	98.470	118.116	96.482
		*α* → −0.5	54.805	45.347	44.723	42.123	44.885	42.212
		*β* → 0.6	176.298	223.978	153.566	165.876	159.602	178.904
		*β* → 0.65	139.513	182.509	130.624	139.055	132.134	147.753
		*η*_*t*_ ∼ *Z*_5_	95.764	603.784	75.170	73.651	76.023	72.775
		*η*_*t*_ ∼ *Z*_7_	50.551	149.586	41.259	39.243	42.111	40.113
			*ρ* = 0.998	*ρ* = 0.9975	*k* = 0.5	*k* = 0.5	λ = 0.2	λ = 0.2
			*κ* = 250	*κ* = 5	*h* = 9.9	*h* = 7.8	*L*_*e*_ = 5.9	*L*_*e*_ = 4.5
*η*_*t*_ ∼ *Z*_1,2_	ARL_0_		370.209	369.133	367.587	371.669	370.751	364.270
	ARL_1_	*ω* → 0.5	179.342	204.354	153.562	164.022	158.206	162.620
		*ω* → 0.7	86.405	113.404	64.217	70.106	67.922	75.622
		*ω* → 1	29.556	51.516	22.845	26.069	23.854	29.478
		*α* → 0.5	325.015	403.328	283.130	293.210	277.024	306.221
		*α* → −0.3	99.949	94.624	91.175	84.102	91.119	80.756
		*α* → −0.5	37.514	37.528	33.915	32.206	34.620	33.268
		*β* → 0.6	175.466	232.909	153.254	163.113	156.983	169.677
		*β* → 0.65	153.682	200.431	137.443	144.553	140.005	152.114
		*η*_*t*_ ∼ *Z*_5_	55.581	193.527	49.379	48.418	49.455	48.468
		*η*_*t*_ ∼ *Z*_7_	32.437	73.613	30.057	29.892	30.212	30.351

**Table 8:** ARLs of control charts when trained with samples obtained with the wild-bootstrap method, where *η*_*t*_ ∼ *N*(0, 1), and no additive outliers are present.

			(*ω*, *α*_1_, *α*_2_, *β*) = (0.3, 0.1, 0.5, 0.3)					
			OCC		CUSUM		EWMA	
			HSVR	GARCH	HSVR	GARCH	HSVR	GARCH
			*ρ* = 0.9938	*ρ* = 0.9995	*k* = 0.5	*k* = 0.5	λ = 0.2	λ = 0.2
			*κ* = 250	*κ* = 50	*h* = 14.2	*h* = 6.9	*L*_*e*_ = 2.8	*L*_*e*_ = 4.1
GJR-GARCH	ARL0		370.622	371.824	364.122	372.010	360.088	355.087
	ARL1	*ω* → 0.5	61.519	115.420	32.787	58.282	314.995	56.296
		*ω* → 0.7	30.811	63.144	16.618	29.066	135.861	29.788
		*ω* → 1	17.799	33.314	10.171	16.154	61.662	17.080
		*α*_1_ → 0.6	43.019	126.849	26.936	48.237	120.102	48.616
		*α*_1_ → 0.9	27.067	62.662	18.947	30.317	56.604	30.828
		*α*_2_ → 1	47.725	90.601	35.594	54.005	133.486	52.625
		*α*_2_ → 1.3	31.444	57.345	23.958	34.161	78.489	34.834
		*β* → 0.6	23.487	46.438	14.646	23.508	70.637	23.955
		*β* → 0.65	15.328	29.615	10.058	15.541	40.632	16.859
		*η*_*t*_ ∼ *Z*_3_	18.395	18.419	15.449	16.344	16.289	17.129
		*η*_*t*_ ∼ *Z*_5_	14.731	14.566	12.832	13.584	13.525	14.097
			(*ω*, *α*, *β*) = (0.3, 0.3, 0.3)					
			*ρ* = 0.9953	*ρ* = 0.999	*k* = 0.5	*k* = 0.5	λ = 0.2	λ = 0.2
			*κ* = 2500	*κ* = 75	*h* = 6.8	*h* = 6	*L*_*e*_ = 3.8	*L*_*e*_ = 3.5
log-GARCH	ARL0		369.721	367.541	367.934	371.064	360.795	378.739
	ARL1	*ω* → 0.5	34.877	524.213	27.419	382.301	28.846	312.913
		*ω* → 0.7	15.970	222.033	13.485	121.000	14.263	111.171
		*ω* → 1	6.739	71.487	6.229	34.558	6.791	34.932
		*α* → 0.5	141.054	493.984	75.104	1654.612	128.860	1720.132
		*α* → 0.65	32.487	19.643	34.113	61.382	1570.849	1976.441
		*β* → 0.5	80.443	757.208	63.678	963.312	66.223	865.125
		*β* → −0.5	92.463	281.624	78.558	241.586	82.630	256.894
		*β* → −0.9	49.150	76.976	51.595	63.780	54.173	67.690
		*η*_*t*_ ∼ *Z*_5_	13.847	6.582	13.043	18.771	13.269	19.132
		*η*_*t*_ ∼ *Z*_7_	11.987	6.129	11.198	15.306	11.715	15.404

### 4.2 Experiment results

In this subsection, we report the results of three experiments as follows. First, we compare the performance of our proposed OCC-based control chart with that of [47] by comparing control charts constructed from either ${\hat{\epsilon }}_{t}^{2}$ or ${\tilde{\epsilon }}_{t}^{2}$, where the latter denotes the residuals employed in [47]. For fairness, we consider the control charts constructed from HSVR-GARCH residuals for both models to avoid the model misspecification issue in this particular experiment. Finally, we present the performance of control charts constructed from wild-bootstrap samples introduced in Remark 1 of Section 3.2. For the latter experiment, we set the number of bootstrap samples as *B* = 1000.

Table 1 report the comparison of OCC, CUSUM, and EWMA control charts that utilize the squared residuals ${\hat{\epsilon }}_{t}^{2}$ against those that uses ${\tilde{\epsilon }}_{t}^{2}$, where the underlying model is GJR-GARCH(1,1) with only innovational outliers of *Z*_1,2_ being present. Note that for OCC-based control charts, directly using ${\hat{\epsilon }}_{t}^{2}$ yields significantly enhanced results compared to those using ${\tilde{\epsilon }}_{t}^{2}$. Moreover, in most circumstances using ${\hat{\epsilon }}_{t}^{2}$, a small improvement is observed regarding the detection ability in the latter two control charts. We speculate that this phenomenon occurred because ${\tilde{\epsilon }}_{t}^{2}$ diluted the information contained in the residuals, therefore undermining the overall detection ability. Indeed, as HSVR-GARCH model possesses the robustness on the estimated conditional volatility, squared residuals also become stabilized, thereby allowing to circumvent the constraint of using heuristic methods that struggle to suppress numerical instabilities. The result also implies that our proposed OCC-based control chart is superior to that of [47] in terms of the ARL_1_ performance.

Tables 2–4 depict the ARLs of control charts when the underlying model is GJR-GARCH model, where the experiment of the latter two tables particularly contains additive outliers of Cases 1 and 4, respectively. The overall performance of charts with HSVR-GARCH residuals is relatively superior especially when the dataset is contaminated with either innovational outliers or additive noises, although the detection ability of HSVR-GARCH-based control charts somewhat recedes when neither of them are present. Specifically, ARL_1_ of CUSUM and EWMA charts with HSVR-GARCH residuals consistently detect a change which is 5-10 percent faster than GARCH(1,1) counterparts. In particular, control charts with HSVR-GARCH residuals commonly exhibit stellar detection ability over their GARCH(1,1) residual counterparts when *α*_*j*_ (*j* = 1, 2) experience a change. This finding validates HSVR-GARCH models to be highly effective in capturing a structural change when the time series is systematically more convoluted.

Furthermore, unlike [47], our OCC-based chart is shown to compare well with the CUSUM and EWMA charts that use identical squared residuals in most cases and to benefit the most from using HSVR-GARCH residuals among the three control charts. Most notably, all results regarding GJR-GARCH model excluding the case of no outliers indicate the loss of the detection ability of the OCC-based control chart when GARCH(1,1) residuals are used. This phenomenon is most likely resulting from the ill-behaved residuals due to the model bias. Therefore, it is practically advised to jointly deploy the OCC-based control charts with the HSVR-GARCH model to avoid this model bias problem.

Tables 5–7 portray the ARL_0_ and ARL_1_ of control charts when time series follows the log-GARCH model. Note that the latter two tables specifically depict the dataset contaminated with additive outliers of Cases 3 and 4, respectively. Here, HSVR-GARCH model-based control charts are observed to be more competent, as they detect a structural change up to twice as fast especially when only innovational outliers are present. Indeed, the effect of model misspecification becomes more apparent as control charts with the parametric GARCH(1,1) residuals lose detection ability in some circumstances like the case of *α* changing from 0.3 to 0.5. Although HSVR-GARCH residuals-based charts do not outperform when the innovations structurally become heterogeneous, HSVR-GARCH residuals still continue to be a superior choice for OCC-based control charts as those residuals significantly shorten ARL_1_ compared to the other two charts, while still providing a stable chart.

In addition, unlike the results of GJR-GARCH models, control charts in log-GARCH models without any external additive outliers is shown to be much more stable and immensely powerful, especially in the cases when *ω* or the innovational distribution of *η*_*t*_ experience a change. In particular, when either *α* or *β* increases to make a time series to be nearly nonstationary, most control charts with GARCH(1,1) residuals are observed to respond relatively poorly or even fail to respond. In contrast, control charts with HSVR-GARCH residuals not only successfully capture a change under those circumstances, and, on average, detect them twice as fast in the case of the parameter changes. In a nutshell, these results all fortify the strength of constructing control charts with HSVR-GARCH residuals when the underlying model is nonlinear and possibly unknown.

Finally, Table 8 denotes the performance of control charts when they are trained with samples generated by the wild-bootstrap method for both HSVR-GARCH and GARCH(1,1) models, when no outliers exist. Although some degree of performance drop is noticeable in certain cases, the three control charts with HSVR residuals retained the strong detection ability across all settings. Bootstrap methods applied to HSVR-GARCH models can be advantageous particularly when ample samples of in-control time series are unavailable. Note, however, that the bootstrap method can be less effective, for example, when the observed time series is impaired with innovational outliers, as the bootstrap samples are generated from iid standard normal innovations. Therefore, in practice, bootstrap methods can be especially beneficial in real-world scenarios when the source of observations do not possess any serious structural noises.

## 5 Real data analysis

This section demonstrates the real-world performance of the control charts that utilizes HSVR-GARCH residuals by analyzing financial indices, namely, the log-returns of the Nasdaq composite index and Korea Composite Stock Price Index (KOSPI). We regard the indices from June 2, 2014 to October 31, 2017 (863 observations) for the former index and from March 2, 2013 to October 31, 2019 (1,512 observations) for the latter as the pre-observed training time series, and regard the subsequent time series to be monitored afterwards. For simplicity, we denote the respective time series “Nasdaq” and “KOSPI”.

**Fig 3:**
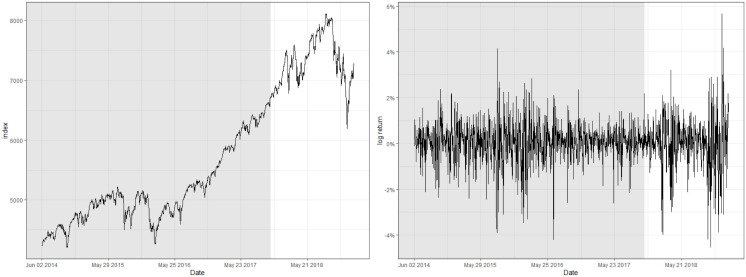
Raw index (left) and its log returns (right) of Nasdaq.

**Fig 4:**
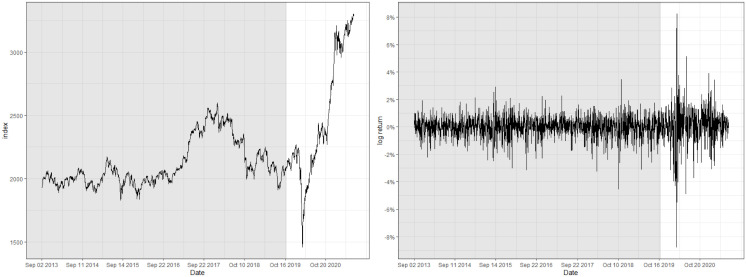
Raw index (left) and its log returns (right) of KOSPI.

**Fig 5:**
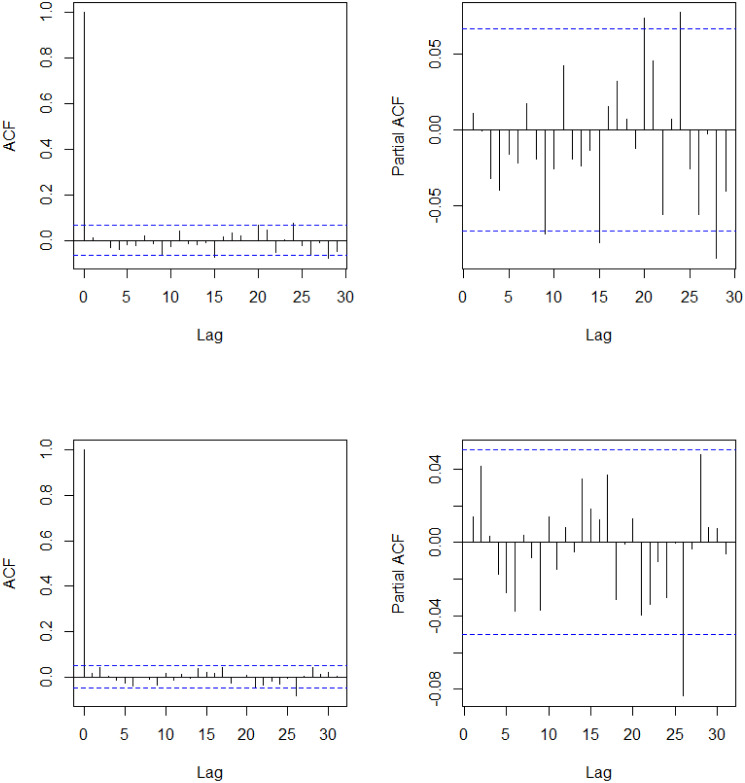
Plot of ACF and PACF up to lag 25 of log-returns for Nasdaq (top) and KOSPI (bottom), respectively.

**Fig 6:**
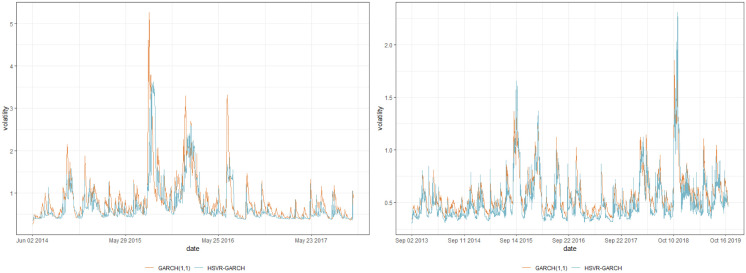
Plot of the predicted conditional volatility for Nasdaq (left) and KOSPI (right) during the training period, respectively. Orange and blue lines respectively denote the conditional volatility estimated via HSVR-GARCH and GARCH(1,1) model.

One of the prominent characteristics of both datasets is that their training set contains a number of abrupt fluctuations, thus is intrinsically unstable. This behavior can be also examined through the indices illustrated in Figs 3 and 4 and Table 9, where the skewness and the excess kurtosis depart from those of a standard normal distribution. Meanwhile, Fig 5 illustrates that both datasets do not contain significant autocorrelations up to lag 12, which validates fitting a pure GARCH-type time series to these datasets. Despite the instability, however, the estimated conditional volatility of HSVR-GARCH models in Fig 6 is observed to resemble the estimates of the GARCH(1,1) model, and the residuals obtained from the former model is revealed to be stable and relatively normally distributed. Moreover, the Ljung-Box test conducted on HSVR-GARCH residuals indicates no significant autocorrelations up to lag 12 on both datasets at the nominal level of 0.05, with *p*-values of 0.0724 and 0.1118, respectively. Most notably, although the training set of Nasdaq seemingly contain a period of the increased volatility, the Ljung-Box test does not reject the null for the residuals of Nasdaq, which is a strong indication that HSVR-GARCH model can successfully capture the conditional volatility in these highly volatile real-world circumstances.

**Table 9:** Summary statistics of the training time series, the result of the Ljung-Box test on HSVR-GARCH residuals, and the location of the structural change regarding Nasdaq and KOSPI.

		Nasdaq	KOSPI
summary statistics	length	863	1,512
	mean	0.0534	0.0052
	median	0.0990	0.0373
	standard deviation	0.9131	0.7521
	minimum	-4.2023	-4.5411
	first quartile	-0.3542	-0.3561
	third quartile	0.5107	0.7521
	maximum	4.1520	3.4728
	skewness	-0.4870	-0.5107
	excess kurtosis	2.5443	2.3162
Ljung-Box Test	Test statistics	19.73	0.0724
	p-value	18.13	0.1118
change location	OCC	Feb/02/18	Jan/28/20
	CUSUM	Feb/05/18	Jan/28/20
	EWMA	Feb/05/18	Jan/28/20

The gist of the procedure regarding the analysis is as follows. We fit HSVR-GARCH model to the given dataset to obtain $\hat{g}$ that estimates the conditional volatility, then compute 1,000 independent copies of bootstrap samples using $\hat{g}$ as presented in Remark 1 of Section 3.2. Afterwards, we sequentially observe the indices by simultaneously using OCC, CUSUM, and EWMA control charts with HSVR-GARCH residuals to monitor a structural change. The decision boundary of the OCC-based control chart and the tuning parameters necessary for CUSUM and EWMA charts are all computed using bootstrap samples and optimized to have the ARL_0_ of 200, rather than 370, because financial time series is highly infeasible to maintain its structural integrity for a long period of time due to various external or international socioeconomic affairs. This implies that it is generally ill-advised to set a large value of ARL_0_ when constructing control charts for real-world financial time series. Therefore, practitioners are suggested to construct plural control charts, avoiding a possible biased result when monitoring financial time series. All unmentioned but remaining settings required for constructing the control charts, such as *δ* in (15), are identical to those of Section 4.

**Fig 7:**
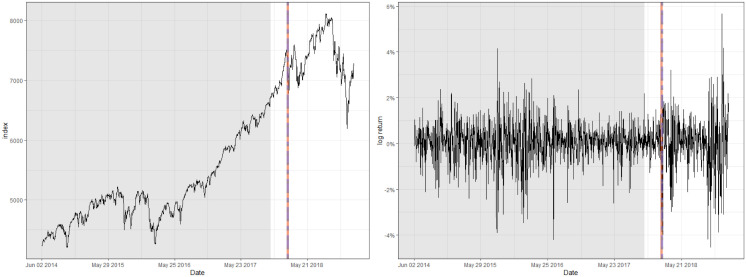
Plot of the detected location of a structural change for Nasdaq. Left and right plots respectively depict the results on the raw index and the log-returns of Nasdaq, while solid, dotted, and dashed lines respectively denote the location of a change for OCC-based, CUSUM, and EWMA charts.

**Fig 8:**
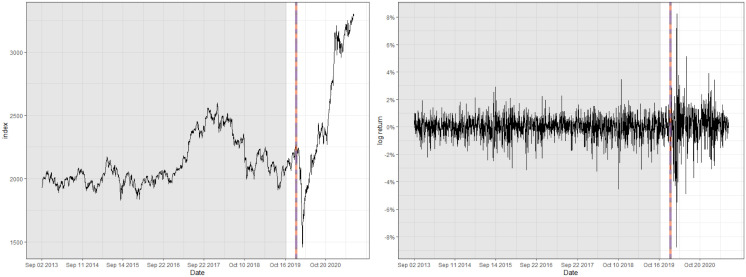
Plot of the detected location of a structural change for KOSPI.

Table 9, alongside with Figs 7 and 8 report the detected location of the change for Nasdaq and KOSPI. For Nasdaq, the optimal set of tuning parameters for OCC, CUSUM, and EWMA charts are respectively computed as (*ρ*, *κ*) = (0.9995, 0.05), (*k*, *h*) = (0.5, 5.3), and (λ, *L*_*e*_) = (0.2, 3.4). Moreover, the detected location of a change for all three charts appear to be similar, namely on February 2, 2018 for the OCC-based chart, and three days later for CUSUM and EWMA charts, respectively.

The period of the potential structural break is around the commencement of the global trade dispute between the United States and China, which started in late January, 2018. This incident can be considered as a decisive factor triggering the abrupt upsurge of the conditional volatility, as both nations continuously imposed retaliatory tariffs over the following months. In particular, the OCC-based control charts that signaled the change three days before the other two charts illuminate that OCC-based control charts, combined with HSVR-GARCH residuals, can be functional in circumstances where promptly notifying a change is critical.

On the contrary, for KOSPI, the optimal set of tuning parameters for OCC, CUSUM, and EWMA charts are obtained to be (*ρ*, *κ*) = (0.9995, 0.025), (*k*, *h*) = (0.5, 5.0), and (λ, *L*_*e*_) = (0.2, 3.1), respectively. Also, analogous to the preceding analysis, all three control charts detected a volatility change on January 28, 2020. Indeed, Fig 4 depicts a noticeable decline of the log-returns after the change point. The date of change is precisely located around when the severity of COVID-19 outbreak was rapidly escalating in China, while the first patient was concurrently reported in South Korea. This finding illuminates that South Korean economy suffered doubly because of COVID-19, as this incident predates the crash of the global stock market due to COVID-19 that occurred in early March, 2020. Moreover, this result further connotes that the global economy actually received a warning preceding the significant collapse in the following months.

## 6 Concluding remarks

This study demonstrated the merits of using residuals obtained from HSVR-GARCH models when formulating control charts, and additionally proposed a significantly improved variant of the OCC-based control chart. Unlike other hybridized GARCH models in the literature, our HSVR-GARCH model uses a likelihood-based loss function to overcome the problem that arises because of the unknownness of true volatility $\sigma_{t}^{2}$. It was observed that the squared residuals computed from the HSVR-GARCH model significantly bolstered the overall detection ability of all control charts including the OCC-based one, and was proven to outperform those using residuals from the parametric GARCH model, especially in circumstances where the underlying model is nonlinear, sophisticated, or contaminated with innovational or additive outliers. The monitoring method combining the squared residuals of the HSVR-GARCH model and the OCC-based control chart consistently and promptly detected a structural change even when the observed time series are heavily contaminated and unstable. We also verified the validity of using bootstrap samples obtained from the HSVR-GARCH model when constructing the OCC-based chart, which can be crucial practical in training control charts when a large amount of in-control time series is not available, which is a usual case in the financial time series analysis.

Despite a number of improvements having been made to the OCC-based control chart, we can claim to facilitate the OCC-based control chart to be even more powerful by providing a higher dimensional training dataset to SVDD, through embedding the time series or its residual process to some adequate lower-dimensional structure-preserving feature space. Due to its importance, this task is worth further investigation and remains our future research project.

## References

[1] Montgomery DC. Introduction to statistical quality control, 7th Ed. John Wiley & Sons; 2012.

[2] Berthouex P, Hunter W, Pallesen L. Monitoring sewage treatment plants: some quality control aspects. Journal of Quality Technology. 1978;10(4):139–149. doi: 10.1080/00224065.1978.11980842

[3] Alwan LC, Roberts HV. Time-series modeling for statistical process control. Journal of business & economic statistics. 1988;6(1):87–95. doi: 10.2307/1391421

[4] Harris TJ, Ross WH. Statistical process control procedures for correlated observations. The canadian journal of chemical engineering. 1991;69(1):48–57. doi: 10.1002/cjce.5450690106

[5] Montgomery DC, Mastrangelo CM. Some statistical process control methods for autocorrelated data. Journal of Quality Technology. 1991;23(3):179–193. doi: 10.1080/00224065.1991.11979321

[6] Alwan LC. Effects of autocorrelation on control chart performance. Communications in statistics-Theory and Methods. 1992;21(4):1025–1049. doi: 10.1080/03610929208830829

[7] Lu CW, Reynolds MR. Control charts for monitoring the mean and variance of autocorrelated processes. Journal of Quality Technology. 1999;31(3):259–274. doi: 10.1080/00224065.1999.11979925

[8] Loredo EN, Jearkpaporn D, Borror CM. Model-based control chart for autoregressive and correlated data. Quality and reliability engineering international. 2002;18(6):489–496. doi: 10.1002/qre.497

[9] Dyer J, Conerly M, Adams BM. A simulation study and evaluation of multivariate forecast based control charts applied to ARMA processes. Journal of Statistical Computation and Simulation. 2003;73(10):709–724. doi: 10.1080/0094965031000062168

[10] Noorossana R, Vaghefi SJM. Effect of autocorrelation on performance of the MCUSUM control chart. Quality and Reliability Engineering International. 2006;22(2):191–197. doi: 10.1002/qre.695

[11] Chang SI, Zhang K. Statistical process control for variance shift detections of multivariate autocorrelated processes. Quality Technology & Quantitative Management. 2007;4(3):413–435. doi: 10.1080/16843703.2007.11673161

[12] Osei-Aning R, Abbasi SA, Riaz M. Mixed EWMA-CUSUM and mixed CUSUM-EWMA modified control charts for monitoring first order autoregressive processes. Quality Technology & Quantitative Management. 2017;14(4):429–453. doi: 10.1080/16843703.2017.1304038

[13] Francq C, Zakoian JM. Maximum likelihood estimation of pure GARCH and ARMA-GARCH processes. Bernoulli. 2004;10(4):605–637. doi: 10.3150/bj/1093265632

[14] Issam BK, Mohamed L. Support vector regression based residual MCUSUM control chart for autocorrelated process. Applied mathematics and computation. 2008;201(1-2):565–574. doi: 10.1016/j.amc.2007.12.059

[15] Cuentas S, Peñabaena-Niebles R, Garcia E. Support vector machine in statistical process monitoring: a methodological and analytical review. The International Journal of Advanced Manufacturing Technology. 2017;91(1):485–500. doi: 10.1007/s00170-016-9693-y

[16] Zhang H, Albin S. Determining the number of operational modes in baseline multivariate SPC data. IIE transactions. 2007;39(12):1103–1110. doi: 10.1080/07408170701291787

[17] Maboudou-Tchao EM. Monitoring the mean with least-squares support vector data description. Gestão & Produção. 2021;28.

[18] Maboudou-Tchao E, Harrison CW, Sen S. A comparison study of penalized likelihood via regularization and support vector-based control charts. Quality Technology & Quantitative Management. 2023;20(2):147–167. doi: 10.1080/16843703.2022.2096198

[19] Vapnik VN. The Nature Of Statistical Learning Theory. New York: Springer; 2000.

[20] Smola A, Schölkopf B. A tutorial on support vector regression. Statistics and computing. 2004;14:199–222. doi: 10.1023/B:STCO.0000035301.49549.88

[21] Fernandez-Rodriguez F, Gonzalez-Martel C, Sosvilla-Rivero S. On the profitability of technical trading rules based on artificial neural networks: evidence from the Madrid stock market. Economics Letters. 2000;69:89–94. doi: 10.1016/S0165-1765(00)00270-6

[22] Cao L, Tay F. Financial forecasting using support vector machines. Neural Computation and Application. 2001;10:184–192. doi: 10.1007/s005210170010

[23] Pérez-Cruz F, Afonso-Rodriguez J, Giner J. Estimating GARCH models using SVM. Quantitative Finance. 2003;3:163–172. doi: 10.1088/1469-7688/3/3/302

[24] Chen S, Härdle WK, Jeong K. Forecasting volatility with support vector machine-based GARCH model. Journal of Forecasting. 2010;433:406–433. doi: 10.1002/for.1134

[25] Bezerra PCS, Albuquerque PHM. Volatility forecasting via SVR–GARCH with mixture of Gaussian kernels. Computational Management Science. 2017;14:179–196. doi: 10.1007/s10287-016-0267-0

[26] Lee S, Lee S, Moon M. Hybrid change point detection for time series via support vector regression and CUSUM method. Applied Soft Computing. 2020;89(106101).

[27] Lee S, Kim C, Lee S. Hybrid CUSUM change point test for time series with time-varying volatilities based on support vector regression. Entropy. 2020;22:578. doi: 10.3390/e22050578 33286350 PMC7517100

[28] Lee S, Kim CK, Kim D. Monitoring volatility change for time series based on support vector regression. Entropy. 2020;22(11):1312. doi: 10.3390/e22111312 33287077 PMC7712961

[29] Kim CK, Lee S. Conditional quantile change test for time series based on support vector regression. Communications in Statistics-Simulation and Computation. 2021; p. 1–18.

[30] Zhang X. Using class-center vectors to build support vector machines. In: Neural Networks for Signal Processing IX: Proceedings of the 1999 IEEE Signal Processing Society Workshop (Cat. No. 98TH8468). IEEE; 1999. p. 3–11.

[31] Mangasarian OL, Musicant DR. Robust linear and support vector regression. IEEE Transactions on Pattern Analysis and Machine Intelligence. 2000;22(9):950–955. doi: 10.1109/34.877518

[32] Zhao Y, Sun J. Robust support vector regression in the primal. Neural Networks. 2008;21(10):1548–1555. doi: 10.1016/j.neunet.2008.09.001 18829255

[33] Balasundaram S, Meena Y. Robust support vector regression in primal with asymmetric Huber loss. Neural Processing Letters. 2019;49(3):1399–1431. doi: 10.1007/s11063-018-9875-8

[34] Sun R, Tsung F. A kernel-distance-based multivariate control chart using support vector methods. International Journal of Production Research. 2003;41(13):2975–2989. doi: 10.1080/1352816031000075224

[35] Tax DM, Duin RP. Support vector data description. Machine learning. 2004;54(1):45–66. doi: 10.1023/B:MACH.0000008084.60811.49

[36] Sukchotrat T, Kim SB, Tsung F. One-class classification-based control charts for multivariate process monitoring. IIE transactions. 2009;42(2):107–120. doi: 10.1080/07408170903019150

[37] Kim SB, Jitpitaklert W, Sukchotrat T. One-class classification-based control charts for monitoring autocorrelated multivariate processes. Communications in Statistics—Simulation and Computation. 2010;39(3):461–474. doi: 10.1080/03610910903480826

[38] Gani W, Limam M. Performance evaluation of one-class classification-based control charts through an industrial application. Quality and Reliability Engineering International. 2013;29(6):841–854. doi: 10.1002/qre.1440

[39] Gani W, Limam M. A one-class classification-based control chart using the-means data description algorithm. Journal of Quality and Reliability Engineering. 2014;2014. doi: 10.1155/2014/239861

[40] Maboudou-Tchao EM. Change detection using least squares one-class classification control chart. Quality Technology & Quantitative Management. 2020;17(5):609–626. doi: 10.1080/16843703.2019.1711302

[41] Vapnik V. Statistical learning theory. New York: John Wiley and Sons; 1998.

[42] Nelson DB. Conditional heteroskedasticity in asset returns: A new approach. Econometrica: Journal of the econometric society. 1991; p. 347–370. doi: 10.2307/2938260

[43] Fung G, Mangasarian OL. Proximal support vector machine classifiers. In: Proceedings of the seventh ACM SIGKDD international conference on Knowledge discovery and data mining; 2001. p. 77–86.

[44] Huang GB, Zhou H, Ding X, Zhang R. Extreme learning machine for regression and multiclass classification. IEEE Transactions on Systems, Man, and Cybernetics, Part B (Cybernetics). 2011;42(2):513–529. doi: 10.1109/TSMCB.2011.2168604 21984515

[45] Schölkopf B, Platt JC, Shawe-Taylor J, Smola AJ, Williamson RC. Estimating the support of a high-dimensional distribution. Neural computation. 2001;13(7):1443–1471. doi: 10.1162/089976601750264965 11440593

[46] Oh H, Lee S. Modified residual CUSUM test for location-scale time series models with heteroscedasticity. Annals of Institute of Statistical Mathematics. 2019;71:1059–1091. doi: 10.1007/s10463-018-0679-4

[47] Lee S, Lee S, Kim CK. One-class classification-based monitoring for the mean and variance of time series. Quality and Reliability Engineering International. 2022;38(5):2548–2565. doi: 10.1002/qre.3090

[48] Chen S, Härdle WK, Jeong K. Forecasting volatility with support vector machine-based GARCH model. Journal of Forecasting. 2010;29(4):406–433. doi: 10.1002/for.1134

[49] Hwang CH, Shin SI. Estimating GARCH models using kernel machine learning. Journal of the Korean Data and Information Science Society. 2010;21(3):419–425.

[50] Liu DC, Nocedal J. On the limited memory BFGS method for large scale optimization. Mathematical programming. 1989;45(1):503–528. doi: 10.1007/BF01589116

[51] Glosten LR, Jagannathan R, Runkle DE. On the relation between the expected value and the volatility of the nominal excess return on stocks. The Journal of Finance. 1993;48:1779–1801. doi: 10.1111/j.1540-6261.1993.tb05128.x

[52] Sucarrat G, Grønneberg S, Escribano A. Estimation and inference in univariate and multivariate log-GARCH-X models when the conditional density is unknown. Computational statistics & data analysis. 2016;100:582–594. doi: 10.1016/j.csda.2015.12.005

[53] Lu CW, Reynolds MR. CUSUM charts for monitoring an autocorrelated process. Journal of Quality Technology. 2001;33(3):316–334. doi: 10.1080/00224065.2001.11980082

[54] Knoth S, Schmid W. Control charts for time series: A review. Frontiers in statistical quality control 7. 2004; p. 210–236. doi: 10.1007/978-3-7908-2674-6_14

[55] Testik MC. Model inadequacy and residuals control charts for autocorrelated processes. Quality and Reliability Engineering International. 2005;21(2):115–130. doi: 10.1002/qre.611

[56] Hansen PR, Lunde A. A forecast comparison of volatility models: does anything beat a GARCH (1, 1)? Journal of applied econometrics. 2005;20(7):873–889. doi: 10.1002/jae.800

